# From Quantum Curves to Topological String Partition Functions

**DOI:** 10.1007/s00220-022-04579-4

**Published:** 2022-12-14

**Authors:** Ioana Coman, Elli Pomoni, Jörg Teschner

**Affiliations:** 1 Deutsches Elektronen-Synchrotron DESY, Notkestrasse 85, 20607 Hamburg, Germany; 2 Department of Mathematics, University of Hamburg, Bundesstrasse 55, 20146 Hamburg, Germany; 3 Institute of Physics, University of Amsterdam, 1098 XH Amsterdam, The Netherlands

## Abstract

This paper describes the reconstruction of the topological string partition function for certain local Calabi–Yau (CY) manifolds from the quantum curve, an ordinary differential equation obtained by quantising their defining equations. Quantum curves are characterised as solutions to a Riemann–Hilbert problem. The isomonodromic tau-functions associated to these Riemann–Hilbert problems admit a family of natural normalisations labelled by the chambers in the extended Kähler moduli space of the local CY under consideration. The corresponding isomonodromic tau-functions admit a series expansion of generalised theta series type from which one can extract the topological string partition functions for each chamber.

## Introduction

Topological string theory on Calabi–Yau (CY) manifolds is a subject which has attracted considerable interest both from theoretical physics and from mathematics. From the point of view of physics, it can provide non-perturbative information on various string compactifications with possible applications to supersymmetric field theories and black hole physics. The subject is mathematically related to various curve counting invariants and to the phenomenon of mirror symmetry. A very fruitful interplay between mathematics and physics on this subject has emerged, with duality conjectures motivated by arguments from theoretical physics suggesting profound and unexpected relations between different parts of mathematics, and mathematical research providing the groundwork for making the ideas from physics sufficiently precise for extracting the relevant predictions, and understanding the theoretical foundations.

A key object in topological string theory is the topological string partition function, mathematically defined as the generating function of the Gromov–Witten invariants. String-theoretic duality conjectures suggest that this partition function is related to the generating functions of the enumerative invariants associated with the names Donaldson–Thomas and Gopakumar–Vafa, respectively. These interpretations do not easily lead to a conceptual characterisation of the topological string partition functions as mathematical objects of their own right, as the relevant generating functions are without further input only defined in the sense of formal series. Various alternative characterisations have been proposed, including matrix models, topological recursion, Chern-Simons theory and the quantisation of the moduli spaces of geometric structures of the relevant families of CY manifolds.

These approaches all have their virtues and drawbacks, as usual, and it seems to us that there is still room for an improvement of our understanding of the topological string partition functions as mathematical objects of their own right. Our paper is an attempt to improve our understanding of the topological string partition functions for a certain class of local CY manifolds. The manifolds *Y* of our interest can be locally described by equations of the form

1.1 $$\begin{aligned} uv-R(x,y)=0, \end{aligned}$$

where *x* and *y* are local coordinates for the cotangent bundle $T^*C$ of a given Riemann surface *C* such that the equation $R(x,y)=0$ defines a covering of *C*. This class of local CY manifolds will be referred to as class $\Sigma$. The local CY in this class are relevant for the description of the ${{\mathcal {N}}}=2$, $d=4$-supersymmetric field theories of class ${{\mathcal {S}}}$ [Ga, GMN09] within string theory by geometric engineering [KKV, KMV], see [DDDHP] or [Sm, Section 3] for more details on the geometry of local CY of class $\Sigma$. Theories of class ${{\mathcal {S}}}$ are labelled by the data $(C,{\mathfrak {g}})$, with *C* being a possibly punctured Riemann surface, and ${\mathfrak {g}}$ a Lie algebra of ADE-type. Our goal is to give a non-perturbative definition of the topological string partition functions for local CY of class $\Sigma$. A subset of the local CY of class $\Sigma$ can be represented by certain limits of toric CY, but such a description does not seem to be known for all CY of class $\Sigma$.

As main example we will consider the case $C=C_{0,4}$, the Riemann sphere with four punctures, and $R(x,y)=y^2-q(x)$, $q(x)(dx)^2$ being a quadratic differential on *C* with regular singularities at the punctures. It corresponds to an $A_1$-theory of class ${{\mathcal {S}}}$ often referred to as the *SU*(2), $N_f=4$ theory. The generalisation to the cases $C=C_{0,n}$ is absolutely straightforward, and the cases where *C* has higher genus or *q* has irregular singularities are certainly within reach. We believe that the resulting picture has a high potential for further generalisations. Covers of higher order corresponding to $A_n$-theories of class ${{\mathcal {S}}}$, for example, can be an interesting next step.

The approach taken here is inspired by the previous work described in [N, OP, LMN, NO, ADKMV], indicating a deep interplay between topological string partition functions, free fermions on algebraic curves, and the theory of classically integrable hierarchies. Our approach can be seen in particular as a concrete realisation of some ideas discussed in [ADKMV] suggesting that a non-commutative deformation of the curve $\Sigma$, often referred to as “quantum curve”, can be used to characterise the topological string partition functions. It seems to us, however, that these ideas have not been realised concretely for the local CY of class $\Sigma$ yet. We will here offer a precise definition of the quantum curves for the cases of our interest, and explain how the quantum curve can be used to define the topological string partition functions.

As mentioned above, one may describe some local CY of class $\Sigma$ as limits of toric CY. Whenever this is possible, one may use the topological vertex [AKMV] to compute the topological string partition functions. However, it is not known how to do this for all local CY of class $\Sigma$. Our goal is to provide a geometric characterisation of topological string partition functions that applies uniformly to the local CY of class $\Sigma$. This characterisation will be checked by comparing with topological vertex computations in the example of $C=C_{0,4}$.

A source of inspiration for us were the works [DHSV, DHS] where it has been argued on the basis of string dualities that there exists a dual description for the topological string in terms of a system of D4 and D6 branes intersecting along the surface $\Sigma$. It can can be argued that the topological string partition functions get represented by the partition functions $Z_{\textrm{ff}}$ of the massless chiral open strings stretching between D4 and D6 branes, defining a system of free fermions on the intersection $\Sigma$. Having a nonzero value of the topological string coupling $\lambda$ corresponds to turning on a B-field on the D6-branes. The effect of the B-field can be described in terms of a non-commutative deformation of $\Sigma$. In [DHS] it has been proposed that in the case of local CY of class $\Sigma$ it is possible to describe the relevant deformation of $\Sigma$ by a differential equation, or equivalently a ${{\mathcal {D}}}$-module, on the underlying base curve *C*. A generalisation of the Krichever correspondence [Kr77a, Kr77b] is proposed in [DHS] leading to a construction of the relevant free fermion partitions $Z_{\textrm{ff}}(\xi ,t;\lambda )$ as Fredholm determinants of certain operators build from the solutions of the differential equation defining the quantum curve, where *t* denotes a collection of parameters for the complex structures of $\Sigma$, while $\xi$ is a tuple of chemical potentials for the free fermion charges. This line of thought leads to the prediction that the topological string partition function $Z_{\textrm{top}}(t,\lambda )$ is related to $Z_{\textrm{ff}}(\xi ,t;\lambda )$ by an expansion of the form

1.2 $$\begin{aligned} Z_{\textrm{ff}}(\xi ,t;\lambda ) =\sum _{p\in H^2(Y,{{\mathbb {Z}}})}e^{p\xi }Z_{\textrm{top}}(t+p\lambda ,\lambda ). \end{aligned}$$

We will in the following refer to series of the form (1.2) as generalised theta series.^1^ This would lead to an elegant mathematical characterisation of the topological string partition function whenever one knows how to define the partition functions of free fermionic field theories on the relevant non-commutative surfaces, and how exactly to extract the topological string partition functions from these objects. The program suggested in [DHSV, DHS] has been realised in some basic examples. Our goal here is to realise it in a case that is sufficiently rich to indicate what needs to be done to generalise this approach to much wider classes of cases.

We will observe two main issues that need to be addressed. It will, on the one hand, be crucial in our approach to allow certain quantum corrections to the equation of the quantum curve represented by terms of higher order in $\lambda$. Straightforward quantisation of the equation $y^2-q(x)=0$ yields a differential equation of Schrödinger type called quantum curve. The resulting equation does not have enough parameters to account for all variables of the free fermion partition functions $Z_{\textrm{ff}}(\xi ,t;\lambda )$. A key new element in our approach is the observation that there are natural quantum corrections one can add to the quantum curve introducing the missing variables, known as apparent singularities. Describing the isomonodromic deformations of the deformed quantum curve in the Hamiltonian formalism leads to the definition of the isomonodromic tau-functions which will turn out to be closely related to the partition functions $Z_{\textrm{ff}}(\xi ,t;\lambda )$.

However, this does not lead to a complete definition of the partition functions $Z_{\textrm{ff}}(\xi ,t;\lambda )$ yet. The definition of free fermion partition functions leaves a normalisation freedom which needs to be fixed. In order to use the quantum curves as an effective instrument for the definition and computation of topological string partition functions one needs to understand how to fix this normalisation freedom.

We will here propose a definition of fully normalised partition functions $Z_{\textrm{ff}}(\xi ,t;\lambda )$ based on choices of distinguished systems of Darboux coordinates for the space of the monodromy data of the quantum curves. The relevant Darboux coordinates are provided by a construction called abelianisation [GMN12, HN]. We will see that there is a natural way to choose such Darboux coordinates for each chamber in the extended Kähler moduli space of the local CY under consideration. This yields a definition of the partition functions $Z_{\textrm{ff}}(\xi ,t;\lambda )$ which is analytic within each chamber, but not analytic along the walls separting different chambers.

The resulting proposal for the definition of $Z_{\textrm{ff}}(\xi ,t;\lambda )$ will in the case $C=C_{0,4}$ be checked against the results of topological vertex computations. By carefully revisiting these computations we find a precise match, chamber by chamber. We find that the non-analytic behaviour of $Z_{\textrm{ff}}(\xi ,t;\lambda )$ across the walls separating different chambers precisely reflects the changes of topological string partition functions induced by flop transitions. This offers substantial support for the conjecture that topological string partition functions can be fully characterised by the systems of Darboux coordinates for the monodromy data of the quantum curves defined by abelianisation.

Some of the techniques we develop for our goals may be of independent interest. This includes the development of a gluing formalism for the free fermion partition functions that are relevant in this context. The gluing formalism establishes a direct connection between Riemann–Hilbert type problems, coordinates for the space of monodromy data, and choices of normalisation for the free fermion partition functions. In this way we can furthermore address the long-standing problem to find natural ways to fix the monodromy-dependence of the isomonodromic tau-functions. The relations to free fermion conformal blocks, and the relation to the Darboux coordinates furnished by abelianisation are found to offer a natural solution to this problem.

We view these results as a step towards a geometric characterisation of the topological string partition functions with the help of the quantum curves. This paper is the first part of a series of papers devoted to this subject. It has been revised after the appearance of [CLT] in order to minimize overlap, and to add some clarifications. Some material from a previous version of this paper has been moved to [CLT].

Let us round off the introduction with some comments on related directions of research, and an application. In the context of Donaldson–Thomas theory for toric CY there is an interesting approach to the emergence of the quantum curve [O09], revealing the origin of the integrable structures of the topological string [OR]. Our goals are different. We use the quantum curve as a key ingredient in a precise description of the topological string partition functions as *analytic objects*. The results can be described as products of certain Fredholm determinants with explicit meromorphic functions. Other approaches to the reconstruction of the topological string partition functions from the quantum curve have been proposed in [ACDKV, GS, GHM, MS].^2^

The precise relation between free fermion partition functions and topological string partition functions established in this paper can be seen as a prediction of the duality conjectures used in [DHSV, DHS]. From a mathematical point of view one may find this relation quite non-obvious. One may, in particular, regard our results as a rather non-trivial quantitative check of the string duality conjectures predicting such relations. We’d ultimately hope that learning to define the topological string partition function non-perturbatively may provide the groundwork for a mathematical understanding of various string dualities.

### Overview

Our goal is to define and calculate the topological string partition functions for the families $$Y_{z,u}$$ of local CY, $$ vw-R(x,y)=0, $$ where $$\Sigma =\Sigma _{z,u}$$ is the double cover of a Riemann surface *C* defined by the equation $$R(x,y)=0$$, where $$R(x,y)=q(x)-y^2$$, $$q(x)(dx)^2$$ being a quadratic differential on *C*. This will be fully worked out in the case $$C=C_{0,4}$$, which is prototypical enough to serve as a guideline for the case of general *C*. The solution will be described in the following steps. Section 2 summarises the relevant features of the geometry of the family $$Y_{z,u}$$ of local CY, and of their mirror manifolds $$X_{z,u}$$ which can be described as certain limits of a family of toric CY.

We then introduce the differential equations defining the quantum curves in Sect. 3. A crucial new ingredient are modifications of the quantum curve by the so-called apparent singularities. This is necessary to account for the variables $\xi$ of the partition functions $Z_{\textrm{ff}}(\xi ,t;\lambda )$. It is explained that this modification can be interpreted as a natural quantum correction that reveals the underlying integrable structures of the problem.

The following Sect. 4 explains how one can associate a free fermion partition function to these differential equations. We demonstrate that the free fermion partition function ${{\mathcal {Z}}}_{\textrm{ff}}$ is proportional to the isomonodromic tau-function ${{\mathcal {T}}}(\mu ;z)$ for the case at hand.

Section 5 introduces a new gluing formalism for the construction of the free fermion partition function ${{\mathcal {Z}}}_{\textrm{ff}}$. It is based on a geometric gluing formalism for Riemann–Hilbert type problems which can be used to define distinguished coordinates for the space of monodromy data. There is a corresponding gluing formalism for free fermion partition functions. It is explained how this gluing formalism can be used to fully fix the normalisation of the partition functions ${{\mathcal {Z}}}_{\textrm{ff}}$, and to compute series expansions for these functions.

In Sect. 6 it is first observed that there exist distinguished coordinates for the monodromy data allowing one to recast the series expansions for ${{\mathcal {Z}}}_{\textrm{ff}}$ in the required form (1.2). A small set of normalisation choices is identified for which this is the case. It is observed that the elements of this set are in one-to-one correspondence with certain coordinates for the moduli space of monodromy data of the quantum curves.

The resulting partition functions ${{\mathcal {Z}}}_{\textrm{ff}}$ are compared to the topological string partition functions computed using the topological vertex in Sect. 7. The partition functions differ from chamber to chamber in the extend Kähler moduli space. Agreement with the fully normalised free fermion partition functions holds if one picks the coordinates defining these functions in a way that depends on the chamber under consideration.

In Sect. 8 it is finally observed that the coordinates that allow one to define partition functions having the required form (1.2) are distinguished in another way: These coordinates can be defined by a construction called abelianisation in the literature [HN]. A simple correspondence is observed between the chambers in the Kähler moduli space in which the partition functions are holomorphic and the networks labelling different coordinate systems. This observation indicates a connection with exact WKB further investigated in the companion paper [CLT].

We conclude in Sect. 9 with a brief summary, a discussion of the role of integrable structures, and some perspectives.

## A Family of Local CY

In this section we will discuss the relevant geometric features of the families of local CY-manifolds studied in the paper. As algebraic varieties one may define the manifolds *Y* by equations of the form

2.1 $$\begin{aligned} vw-R(x,y)=0, \end{aligned}$$

where *R*(*x*, *y*) is a polynomial in two variables. Important geometric features of *Y* are encoded in the curve $\Sigma$ defined by the equation $R(x,y)=0$. Families of curves $\Sigma$ define families $Y\equiv Y_{\Sigma }$ of local CY via (2.1).

### Curves

We will mainly focus our attention on the family $Y_{u,z}\equiv Y_{\Sigma _{u,z}}$ of local CY associated to the family of curves $\Sigma _{u,z}$ defined as

2.2 $$\begin{aligned} \begin{aligned}&\Sigma _{u,z}= \big \{\,(x,y)\in T^*C;\, y^2 = q(x)\,\big \},\\&q(x)=\frac{a_1^2}{x^2} + \frac{a_2^2}{(x-z)^2} + \frac{a_3^2}{(x-1)^2} + \frac{\kappa }{x(1-x)}+ \frac{z(z-1)}{x(x-1)} \frac{u}{(x-z)}, \end{aligned} \end{aligned}$$

with $\kappa = a_1^2 + a_2^2 + a_3^2 - a^2_{4}$. It has a complex two-dimensional moduli space parameterised by the complex variables *z* and *u*. We will see below that the defining equation for $\Sigma _{u,z}$ can be brought into the form $R(x,v)=0$ with a polynomial *R*(*x*, *v*) by a change of coordinates $v=v(x,y)$. The curve $\Sigma _{u,z}$ is a two-fold covering of the four-punctured sphere $C_z\equiv C_{0,4}={\mathbb {P}}^1{\setminus }\{0,z,1,\infty \}$. The variable *u* determines how $\Sigma$ covers the base curve $C_z$, in particular the positions of the four branch points.

The description simplifies in a useful way in the limit $z\rightarrow 0$ corresponding to a degeneration of the base curve $C_z$. Let $\gamma _{s}$ be the cycle on $C_z$ that is pinched when $z\rightarrow 0$, and let ${\hat{\gamma }}_{s}$ be a lift of $\gamma _{s}$ to $\Sigma _{u,z}$ which is odd under the involution exchanging the sheets. We will be interested in degenerations keeping the period of the canonical differential *ydx* along ${\hat{\gamma }}_{s}$ finite for $z\rightarrow 0$. This will be the case if we consider families $(z,u_z)$ such that $u_z=\frac{1}{z}(a^2-a_1^2-a_2^2)$, with $a\in {{\mathbb {C}}}$ finite. Indeed, setting $u=\frac{1}{z}(a^2-a_1^2-a_2^2)$ in (2.2), it is straightforward to see that the region on $\Sigma _{u,z}$ with $x={{\mathcal {O}}}(1)$ for $z\rightarrow 0$ can be approximately represented by the branched cover $\Sigma _{\textrm{out}}$ of $C_{0,3}={\mathbb {P}}^1{\setminus }\{0,1,\infty \}$ defined by the equation

2.3 $$\begin{aligned} y^2=\frac{x^2 a_4^2-x(a^2+a_4^2-a_3^2)+a^2}{x^2(x-1)^2}. \end{aligned}$$

From (2.3) is easy to see that the integral $\int _{{\hat{\gamma }}_s}ydx$ is proportional to *a*, as required.

The region in $\Sigma _{u,z}$ with $x=tz$, with *t* finite when $z\rightarrow 0$, may be represented as another branched cover $\Sigma _{\textrm{in}}$ of $C_{0,3}$, defined by

2.4 $$\begin{aligned} (zy)^2=\frac{t^2 a^2-t(a^2+a_1^2-a_2^2)+a^2_1}{t^2(t-1)^2}. \end{aligned}$$

We see that $\Sigma _{u,z}$ degenerates into the union of $\Sigma _{\textrm{out}}$ and $\Sigma _{\textrm{in}}$ for $z\rightarrow 0$. The parameter *a* determining the behaviour of the parameter *u* in the degeneration of $\Sigma _{u,z}$ is found to describe the singular behaviour at the points of $\Sigma _{\textrm{out}}$ and $\Sigma _{\textrm{in}}$ corresponding to the double point on $\Sigma _{u,z}$ arising in the degeneration.

**Fig. 1:**
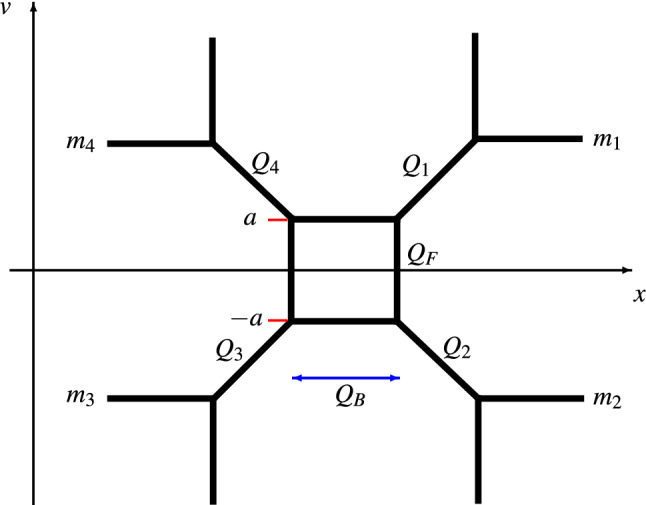
The toric graph of the mirror $X_{R;U,z}$ to the local CY $Y_{R;U,z}$

### Four-dimensional limit and local mirror symmetry

It will later be useful to recall that the family of curves $\Sigma _{u,z}$ can be represented as the limit $R\rightarrow 0$ of a certain family of curves $\Sigma _{R;U,z}$ in ${{\mathbb {C}}}^*\times {{\mathbb {C}}}^*$ related by mirror symmetry to the family of toric Calabi–Yau manifolds^3^ having the toric graph depicted in Fig. 1. The Kähler parameters $t_1,\dots ,t_4,t_F, t_B$ of the toric Calabi–Yau manifolds will be parameterised through the variables $Q_i=e^{-t_i}$, $i=1,\dots ,4$, $Q_F=e^{-t_F}, Q_B=e^{-t_B}$ assigned to the edges of the toric graph in Fig. 1.

We will consider a certain scaling limit of the Kähler parameters which has been used for the geometric engineering [KKV, KMV] of the four-dimensional, ${{\mathcal {N}}}=2$ supersymmetric gauge theory with gauge group *SU*(2) and four flavors within string theory, see e.g. [HIV] for a review discussing this case. The relevant limit, in the following referred to as four-dimensional (4d) limit, is most easily defined by parameterising the Kähler parameters $t_1,\dots ,t_4,t_F, t_B$ as

2.5 $$\begin{aligned} \begin{aligned}&t_{1}= {R(m_1-a)},\\&t_{2}= {R(- a-m_2)}, \end{aligned}\qquad \begin{aligned}&t_{3}={R(- a-m_3)},\\&t_{4}={R(m_4-a)}, \end{aligned} \qquad t_{F}= {2Ra}, \end{aligned}$$

and sending $R\rightarrow 0$. To simplify the exposition we will assume that $m_i\in {{\mathbb {R}}}$ for $i=1,\dots ,4$. In (2.5) we are anticipating a parameterisation which will turn out to be useful later. It is based on the fact that the Kähler parameter associated to an edge with equation $rx+sv=c$ and length *l* is simply given as $l/\sqrt{r^2+s^2}$. Applying this rule to the toric graph in Fig. 1 gives a direct relation between the parameters $m_i\in {{\mathbb {R}}}$, $i=1,\dots ,4$, in (2.5) and the values of the coordinate *v* of the corresponding horizontal external edges indicated in Fig. 1.

Local mirror symmetry [CKYZ] relates this family of toric CY to a family of local CY denoted by $\Sigma _{R;U,z}$. Based on the duality with brane constructions it has been argued in [BPTY]^4^ that the curves $\Sigma _{R;U,z}$ can be defined by the equations

2.6 $$\begin{aligned}&(w-M_1)(w-M_2) x^2 \nonumber \\&\quad -\left( \left( { M_1 M_2 }\right) ^{\frac{1}{2}} \left[ \big (1 +zM^{-\frac{1}{2}}\big )w^2 +\big (1 +zM^{+\frac{1}{2}}\big ) \right] - U w \right) x \nonumber \\&\quad + z \left( \frac{ M_1 M_2 }{ M_3 M_4 } \right) ^{\frac{1}{2}} (w-M_3)(w-M_4) = 0. \end{aligned}$$

We are using the notation $M=M_1M_2M_3M_4$. Considering fixed values for $M_1,\dots ,M_4$, we will regard the two variables *z* and *U* as parameters for the family of curves $\Sigma _{R;U,z}$. The parameters $M_1,\dots ,M_4,U,z$ of the curve defined by the equation (2.6) are related to the Kähler parameters by the mirror map, expressing $t_1,\dots ,t_4,t_F, t_B$ as periods of the canonical one-form $\lambda =\log (w) d\log (x)$ along a suitable set of cycles. The rules of local mirror symmetry imply a simple relation between the parameters $M_1,\dots ,M_4$ in (2.6) and the parameters $m_1,\dots ,m_4$ introduced via (2.5), $M_i=e^{-Rm_i}$ for $i=1,\dots ,4$. Indeed, it is easy to see that $x\rightarrow \infty$ implies that the coordinate $v=-\frac{1}{R}\log (w)$ must approach one of the values $v=m_1$ or $v=m_2$, and similarly for $x\rightarrow 0$. The relation between the parameters *U*, *z* in (2.6) and the parameters $t_B$, $t_F=2Ra$ is more complicated. There exists cycles $\gamma _B$ and $\gamma _F$ on $\Sigma _{R;U,z}$ allowing us to represent the parameters $t_B$ and $t_F$ as the periods $t_B=\int _{\gamma _B}\lambda$ and $t_F=\int _{\gamma _F}\lambda$, respectively.

As discussed in detail in Appendix B of [BPTY], taking the limit $R\rightarrow 0$ of the equation (2.6) with *w* being of the form $w=e^{-Rv}$ yields the following equation

2.7 $$\begin{aligned} \begin{aligned}&(v - m_1) (v - m_2) x^2+ \left( -(1+z)v^{2} + z (m_1+m_2+m_3+m_4) v + h\right) x\\&\quad + z (v - m_3) (v - m_4) = 0, \end{aligned} \end{aligned}$$

with parameter *h* being related to the higher order terms in the expansion of *U* in powers of *R*. This curve can be identified with the curve defined in (2.2) by the change of coordinates $(x,v)\rightarrow (x,y)$ defined by

2.8 $$\begin{aligned} xy = v - \frac{P_1(x)}{2(x-1)(x-q)} ~,\quad P_1(x) = (m_1+m_2)x^2 -z \, {\bar{m}}\,x +z (m_3+m_4), \end{aligned}$$

with ${\bar{m}}=m_1+m_2+m_3+m_4$, bringing the equation for the curve to the form

2.9 $$\begin{aligned} y^2 = \frac{P_1^2(x) - 4(x-1)(x-z)P_2(x)}{4x^2(x-1)^2(x-z)^2}, \qquad P_2(x) = m_1m_2 x^2 +h\,x + z m_3m_4.\qquad \end{aligned}$$

This is easily recognised as the curve (2.2), with

2.10 $$\begin{aligned}&m_4-m_3 = 2a_4, \quad m_4+m_3 = 2a_3, \quad m_1-m_2 = 2a_1, \quad m_1+m_2 = 2a_2, \end{aligned}$$

assuming a certain relation between *h* and *u* that won’t be needed in the following.

### Extended Kähler moduli space

It will be important for us to notice that only a part of the moduli space of the complex structures of $\Sigma _{R;U,z}$ is covered by the mirror duals of the toric CY having the toric graph depicted in Fig. 1. To cover the full moduli space of complex structures one will need other toric CY, related to the one considered above by flop transitions. We may introduce an extended Kähler moduli space which can be described as a collection of chambers representing the Kähler moduli spaces of all toric CY having a mirror dual of the same topological type, joined along walls associated to flop transitions.

Our next goal is to describe the chamber structure of the extended Kähler moduli space in the case $R\rightarrow 0$ of our main interest. It is instructive to first analyse the situation in the limit $z\rightarrow 0$ where $\Sigma _{u,z}$ can be described as the union of $\Sigma _{\textrm{out}}$ and $\Sigma _{\textrm{in}}$. The curves $\Sigma _{\textrm{in}}$ and $\Sigma _{\textrm{out}}$ are determined by the parameters $a^2$, $a_i^2$, $i=1,\dots ,4$. We get an unambiguous parameterisation assuming ${\textrm{Re}}(a)\ge 0$ and ${\textrm{Re}}(a_i)\ge 0$, $i=1,\dots ,4$. The equation for $\Sigma _{\textrm{in}}$ can be written as

2.11 $$\begin{aligned} y^2t^2(t-1)^2=a^2\left( t-\frac{a^2+a_1^2-a_2^2}{2a^2}\right) ^2-\frac{D(a)}{4a^2}, \end{aligned}$$

with $D(a)=(a+a_1+a_2)(a-a_1-a_2)(a+a_1-a_2)(a-a_1+a_2).$ In the case $a_1>a_2$ we see that there exist three chambers,

2.12 $$\begin{aligned}&{\mathfrak {C}}^{\textrm{in}}_{{{\mathfrak {1}}}}=\{\,a\in {{\mathbb {C}}}\,;\, {\textrm{Re}}(a)\ge 0\,, a_1-a_2>{\textrm{Re}}(a)\,\},\end{aligned}$$

2.13 $$\begin{aligned}&{\mathfrak {C}}^{\textrm{in}}_{{{\mathfrak {2}}}}=\{\,a\in {{\mathbb {C}}}\,;\, {\textrm{Re}}(a)\ge 0\,, a_1-a_2<{\textrm{Re}}(a)< a_1+a_2\,\},\end{aligned}$$

2.14 $$\begin{aligned}&{\mathfrak {C}}^{\textrm{in}}_{{{\mathfrak {3}}}}=\{\,a\in {{\mathbb {C}}}\,;\, {\textrm{Re}}(a)\ge 0\,, {\textrm{Re}}(a)> a_1+a_2\,\}. \end{aligned}$$

The boundaries of the chambers correspond to zeros of *D*(*a*). Vanishing of *D*(*a*) implies that the two branch points of the covering $$\Sigma _{\textrm{in}}\rightarrow C_{0,3}$$ coalesce. We may note, on the other hand, that it follows from (2.5) and (2.10) that $$ t_{1}= {R(a_2+a_1-a)}$$ and $$t_{2}= {R(a_1- a-a_2)}. $$ Vanishing of *D* is therefore equivalent to the vanishing of a Kähler parameter. The case where $${\textrm{Re}}(t_i)>0$$ for $$i=1,2$$ corresponds to the chamber $${\mathfrak {C}}^{\textrm{in}}_{{{\mathfrak {1}}}}$$.

A similar decomposition into chambers can be introduced for the parameter space of $\Sigma _{\textrm{out}}$. Taken together we arrive at a decomposition of the extended Kähler moduli space of $\Sigma _{u,z}$ for $z\rightarrow 0$ into nine chambers denoted ${\mathfrak {C}}_{{\mathfrak {i}},{\mathfrak {j}}}$, with ${\mathfrak {i}}={{\mathfrak {1}}},{{\mathfrak {2}}},{{\mathfrak {3}}}$ labelling the chambers of $\Sigma _{\textrm{out}}$, and ${\mathfrak {j}}={{\mathfrak {1}}},{{\mathfrak {2}}},{{\mathfrak {3}}}$ labelling the chambers of $\Sigma _{\textrm{in}}$.

**Fig. 2:**
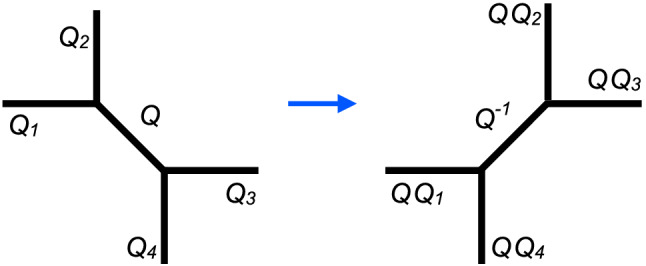
Representation of the flop transition on a subgraph of a toric graph

The resulting qualitative picture can be expected to hold more generally at least in some neighbourhood of the boundary component corresponding to the degeneration $z\rightarrow 0$. The Kähler parameters $t_i$, $i=1,\dots ,4$ can be represented as periods of the canonical one-form along cycles surrounding suitable pairs of branch points. Coalescence of the branch points implies vanishing of the corresponding periods. When one of the periods corresponding to a Kähler parameter $t_i$ becomes negative, one can no longer represent the mirror of the curves $\Sigma _{R;U,z}$ as the toric CY having the graph in Fig. 1. The mirror of $\Sigma _{R;U,z}$ may instead be represented by another toric graph obtained from the one in Fig. 1 by the local modification depicted in Fig. 2. This transition is often called a flop. In Fig. 2 we have also indicated the choice of Kähler parameters on the toric graph related to the original one by a flop. For the case at hand it is easy to verify that the rule indicated in Fig. 2 is necessary to preserve the values of $m_i$ in Fig. 1.

At least in the case where *z* is sufficiently small, we expect to get all relevant toric graphs by applying flops to the toric CY having the toric graph depicted in Fig. 1.

## Quantum Curves, ${{\mathcal {D}}}$-Modules and Integrability

One of the main ideas in [DHSV, DHS] is to regard the relevant free fermion partition functions as deformations of the chiral free fermion partition functions on the curves $\Sigma$ generated by turning on a B-field proportional to $\lambda$ on the D6-branes. The deformation induces a non-commutativity of the coordinates (*x*, *y*), turning the curves $\Sigma$ into objects called quantum curves described by certain ordinary differential equations. We are later going to formulate a precise proposal how to associate a free fermion partition function to a quantum curve. In this section we will explain what a quantum curve is, and why it is natural to allow for quantum corrections in the definition of the quantum curve represented by terms of higher order in $\lambda$.

In Subsection 3.1 below we will observe that the limit $\lambda \rightarrow 0$ has a natural relation to the Hitchin integrable system. The relevant quantum corrections are basically determined by the requirement to have a consistent deformation of the integrable structure that is present at $\lambda =0$, which will be briefly reviewed in 3.1. A general discussion of the differential equations representing the non-commutative deformation of $\Sigma$ is given in Sect. 3.2. It is observed that the moduli space of holomorphic connections on *C* is a natural one-parameter deformation of the Hitchin system. The moduli space of flat holomorphic connections has an equivalent representation as the moduli space of the second order differential operators representing the quantum curves if one allows quantum corrections in the quantum curve containing apparent singularities. The integrable flows of the Hitchin system get “deformed” into the isomonodromic deformation flows. These flows can be represented as motions of the positions of the apparent singularities, which is how the $\lambda$-deformed integrable structure of the Hitchin system is represented by quantum corrected quantum curves.

To simplify the exposition we will mostly restrict to the case of surfaces *C* of genus 0 from now on. It is, however, not hard to generalise the following discussion to curves *C* of higher genus.

### Relation to the Hitchin system

To motivate our proposal let us revisit the case $\lambda =0$, recalling that the chiral free fermion partition functions on $\Sigma$ can be represented as theta functions [AMV], schematically

3.1 $$\begin{aligned} Z_{\Sigma }({\underline{\vartheta }},{\textbf{u}})=\sum _{{\textbf{n}}}e^{{\textrm{i}}\,{\textbf{n}}\cdot {{\underline{\vartheta }}}} \,e^{\frac{{\textrm{i}}}{2}\,{\textbf{n}}\cdot \tau ^{\Sigma }({\textbf{u}})\cdot {\textbf{n}}}e^{{{\mathcal {F}}}_1({\textbf{u}})}. \end{aligned}$$

The tuples of integers ${\textbf{n}}$ represent the fermion fluxes through cycles of $\Sigma$, and $\tau ^{\Sigma }({\textbf{u}})$ is the period matrix of $\Sigma \equiv \Sigma _{{\textbf{u}}}$. The variables ${\underline{\vartheta }}$ in (3.1) are naturally interpreted as coordinates on the Jacobian of $\Sigma$ parameterising degree zero line bundles ${{\mathcal {L}}}$ on $\Sigma$. The free fermion partition function $Z_{\Sigma }({\underline{\vartheta }},{\textbf{u}})$ is thereby recognised as a function of the pair of data $(\Sigma ,{{\mathcal {L}}})$. It provides a local description of a section of a holomorphic line bundle on the Jacobian fibration over the base manifold ${{\mathcal {B}}}$ with coordinates ${\textbf{u}}$ parameterising the complex structures of $\Sigma$.

Such Jacobian fibrations naturally arise in the theory of Hitchin systems [Hi] studying Higgs pairs $({\mathcal {E}},\varphi )$ consisting of a holomorphic bundle ${{\mathcal {E}}}$ and an element $\varphi \in H^0(C,{\textrm{End}}({{\mathcal {E}}})\otimes K_C)$ modulo gauge transformations. The integrability of the Hitchin system is realised through the one-to-one correspondence between Higgs pairs and pairs $(\Sigma ,{\mathcal {L}})$, where $\Sigma$ is the spectral curve,

3.2 $$\begin{aligned} \Sigma \,=\,\{\,(x,y)\in T^*C;\,{\textrm{det}}(y\,{\textrm{id}}-\varphi (x))=0\,\}, \end{aligned}$$

and ${\mathcal {L}}$ is the line bundle on $\Sigma$ of degree zero having fibres which can be identified with the one-dimensional space spanned by an eigenvector of $\varphi$. Conversely, given a pair $(\Sigma ,{{\mathcal {L}}})$, where $\Sigma \subset T^*C$ is a double cover of *C*, and ${{\mathcal {L}}}$ a holomorphic line bundle on $\Sigma$, one can recover $({{\mathcal {E}}}, \varphi )$ via $({{\mathcal {E}}},\varphi ) =(\pi _*({{\mathcal {L}}}), \pi _*(y))$, where $\pi$ is the covering map $\Sigma \rightarrow C$, and $\pi _*$ is the direct image.

To make this construction more explicit, let us consider the case of holomorphic ${\textrm{SL}}(2)$-bundles ${{\mathcal {E}}}$, and introduce a suitably normalised eigenvector $\Phi (x)$ of $\varphi (x)$ called Baker-Akhiezer function. It can locally be represented as

3.3 $$\begin{aligned} \Phi (x)=\frac{1}{\varphi _0+y}\bigg (\begin{matrix} \varphi _0-y \\ \varphi _- \end{matrix} \bigg ), \end{aligned}$$

where $y=y(x)$ is the eigenvalue satisfying $y^2=q(x)$, $q(x)=\varphi _0^2+\varphi _+\varphi _-$. The Baker-Akhiezer function $\Phi (x)$ defined in this way has zeros at the points ${\hat{x}}_k$ projecting to a zero $x_k$ of $\varphi _-$ where furthermore $\varphi _0=y$, and poles at ${\check{x}}_k=\sigma ({\hat{x}}_k)$, with $\sigma$ being the sheet involution. The divisor ${\mathbb {D}}=\sum _{k}({\hat{x}}_k-{\check{x}}_k)$ characterises the line bundle ${{\mathcal {L}}}$.

To further simplify the exposition let us now restrict attention to the case where the surface *C* has genus zero $g=0$ with *n* punctures at $z_1,\dots ,z_n$. The Hitchin system will then coincide with the Gaudin model. The quadratic differential *q*(*x*) defining the curve $\Sigma$ has the form

3.4 $$\begin{aligned} q(x)=\sum _{r=1}^n\left( \frac{a_r^2}{(x-z_r)^2}+\frac{H_r}{x-z_r}\right) . \end{aligned}$$

Fix a canonical basis $\{\alpha _1,\dots ,\alpha _{n-3},\beta _1,\dots ,\beta _{n-3}\}$ for $H_1(\Sigma )$. The periods of *ydx* along $\alpha _k$, $k=1,\dots ,n-3$, give local coordinates $a^k$ for ${{\mathcal {B}}}$. The Abel-map of the divisor ${\mathbb {D}}$,

3.5 $$\begin{aligned} \vartheta _l=\int _\gamma \omega _l, \end{aligned}$$

with $\{\omega _l;l=1,\dots ,n-3\}$ being a basis for $H^1(\Sigma ,K)$ such that $\int _{\alpha _k}\omega _l=\delta _{kl}$, and $\gamma$ in (3.5) being a one-dimensional chain^5^ such that $\partial \gamma ={\mathbb {D}}$, provides coordinates on the Jacobian parameterising the choices of the line bundle ${{\mathcal {L}}}$. The coordinates $({\textbf{a}},{\underline{\vartheta }})$, ${\textbf{a}}=(a_1,\dots ,a_{n-3})$, ${\underline{\vartheta }}=(\vartheta _1,\dots ,\vartheta _{n-3})$ are action-angle coordinates for the Hitchin system. There exists a locally defined function ${{\mathcal {F}}}({\textbf{a}})$ allowing us to express the periods $a_k^{\mathrm{\scriptscriptstyle D}}$ along the dual cycles $\beta _k$ as $a_k^{\mathrm{\scriptscriptstyle D}}=\frac{\partial }{\partial a^k}{{\mathcal {F}}}({\textbf{a}})$. The period matrix $\tau ^{\Sigma }$ is obtained from ${{\mathcal {F}}}$ as $\tau _{kl}^{\Sigma }=\frac{\partial ^2}{\partial a^k\partial a^l}{{\mathcal {F}}}({\textbf{a}})$.

Another useful description of the integrable structure of the Hitchin system uses the pairs $(x_k,y_k)$, with $y_k=y(x_k)$ for $k=1,\dots ,n-3$ as coordinate functions. This description, often referred to as the Separation of Variables (SoV) representation,^6^ represents the phase space as the symmetric product $(T^*C)^{[n-3]}$ with Darboux coordinates $(x_k,y_k)$, $k=1,\dots ,n-3$.

It is worth noting that such Jacobian fibrations arise very naturally in the context of local CY of the type considered in this paper. For the case of compact base curves *C* it has been shown in [DDDHP, DDP] that the corresponding Jacobian fibrations are isomorphic to the intermediate Jacobian fibrations of the associated family $Y_{\Sigma }$ of local CY.

### From quantum curves to ${{\mathcal {D}}}$-modules

In [DHSV, DHS] it is argued that turning on a B-field on the D6-branes induces a non-commutative deformation of the algebra of functions on $\Sigma$ described in terms of the coordinates (*x*, *y*) by the commutation relations $[x,y]={\textrm{i}}\lambda$. The deformed algebra of functions can naturally be identified with the Weyl algebra of differential operators with generators *x* and $-{\textrm{i}}\lambda \partial _x$. It seems natural to describe the resulting deformation of the curve $\Sigma$ with the help of a deformed version of the equation $y^2-q(x)=0$ defining $\Sigma$ which is obtained by replacing *y* by $-{\textrm{i}}\lambda \partial _x$. The equation of the curve $\Sigma$ gets replaced by the differential equation

3.6 $$\begin{aligned} (\lambda ^2\partial _x^2+q(x))\chi =0. \end{aligned}$$

A useful framework for making these ideas precise is provided by the theory of ${{\mathcal {D}}}$-modules.

#### ${{\mathcal {D}}}$-modules, differential equations and flat connections

We will now introduce the basic notions of the theory of ${{\mathcal {D}}}$-modules, and later explain why it is consistent with the point of view of [DHS] to allow certain quantum corrections to the quantum curve obtained by canonical quantisation of the equation for the classical curve $\Sigma _{u,z}$.

A ${{\mathcal {D}}}$-module is a sheaf of left modules over the sheaf ${{\mathcal {D}}}_V$ of differential operators on a smooth complex algebraic variety *V*. For each open subset $U\subset V$ we are given a module ${{\mathcal {F}}}(U)$ over ${{\mathcal {D}}}(U)$, the algebra of differential operators on *U*. The various modules ${{\mathcal {F}}}(U)$ attached to subsets *U* satisfy the compatibility conditions defining a sheaf.

An important class of ${{\mathcal {D}}}$-modules is associated to systems of differential equations. Let ${{\mathcal {G}}}_V$ be a sub-algebra of the algebra ${{\mathcal {D}}}_V$ of differential operators on *V*, generated by commuting differential operators ${{\mathcal {D}}}_i$, $i=1,\dots ,m$. To the system of differential equations

3.7 $$\begin{aligned} {{\mathcal {D}}}_i\Psi =0,\qquad i=1,\dots ,m, \end{aligned}$$

one may associate the ${{\mathcal {D}}}$-module

3.8 $$\begin{aligned} \Delta _{{{\mathcal {G}}}_V}^{}:={{\mathcal {D}}}_V/({{\mathcal {D}}}_V\cdot {{\mathcal {G}}}_V). \end{aligned}$$

A solution $\Psi$ of the system (3.7) defines a ${{\mathcal {D}}}$-module homomorphism sending $1\in \Delta _{{{\mathcal {G}}}_V}^{}$ to $\Psi$. Conversely, having a ${{\mathcal {D}}}$-module homomorphism from $\Delta _{{{\mathcal {G}}}_V}^{}$ to a sheaf ${{\mathcal {F}}}$ one gets a solution $\Psi$ to (3.7) with $\Psi \in {{\mathcal {F}}}$ as the image of $1\in \Delta _{{{\mathcal {G}}}_V}^{}$. The discussion above suggests that we are looking for ${{\mathcal {D}}}$-modules of this type, with ${{\mathcal {G}}}_V$ being generated by a single differential operator ${{\mathcal {D}}}_q$ of the form ${{\mathcal {D}}}_q=\lambda ^2\partial _x^2+q(x)$.

One may note, on the other hand, that another simple type of ${{\mathcal {D}}}$-module is the sheaf of sections of a complex vector bundle ${{\mathcal {E}}}$ on *V* with a holomorphic flat $\lambda$-connection^7^$\nabla _\lambda$. The $\lambda$-connection $\nabla _\lambda$, locally represented as

3.9 $$\begin{aligned} \nabla _\lambda =\lambda \partial _x+\varphi (x),\qquad \varphi =\bigg (\begin{matrix} \varphi _0 &{} \;\;\varphi _+ \\ \varphi _- &{} -\varphi _0\end{matrix}\bigg ), \end{aligned}$$

with $\varphi _0,\varphi _\pm$ holomorphic on *C*, defines the action of the differential operators in ${{\mathcal {D}}}(U)$ on the sections of ${{\mathcal {E}}}$. The ${{\mathcal {D}}}$-modules defined from pairs $({{\mathcal {E}}},\nabla _\lambda )$ can be regarded as natural $\lambda$-deformations of the Higgs pairs $({{\mathcal {E}}},\varphi )$.

Within the moduli space ${{\mathcal {M}}}_{\textrm{flat}}(C)$ of pairs $({{\mathcal {E}}},\nabla _\lambda )$ there is a half-dimensional subspace represented by $\lambda$-connections which are gauge equivalent to $\lambda$-connections of the form

3.10 $$\begin{aligned} \nabla _{\mathrm{\scriptscriptstyle Op}}=\lambda \partial _x+\bigg (\begin{matrix} 0 &{} q \\ 1 &{} 0\end{matrix}\bigg ). \end{aligned}$$

Flat connections of this form are called opers. The horizontality condition $$\nabla _{\mathrm{\scriptscriptstyle Op}}\big ({\begin{matrix} \chi _1 \\ \chi _2\end{matrix}}\big ) =0$$ implies that $\chi _2$ solves the equation ${{\mathcal {D}}}_q\chi _2=0$, and that $\chi _1=\partial _x\chi _2$.

Looking for a deformed version of the free fermion partition function associated to quantum curves one may note that the ${{\mathcal {D}}}$-modules defined by opers only depend on half as many variables as the function $Z_{\Sigma }({\underline{\vartheta }},{\textbf{u}})$ does. The ${{\mathcal {D}}}$-modules associated to pairs $({{\mathcal {E}}},\nabla _\lambda )$, on the other hand, depend on just the right number of variables.

#### Opers with apparent singularities

We are now going to observe that allowing certain quantum corrections in the defining equations produces quantum curves in a natural one-to-one correspondence to flat connections. To this aim we will use the fact that any holomorphic connection is gauge equivalent to an oper connection away from certain singularities of a very particular type which may occur at a collection of points $x_k\in C_{0,n}$, $k=1,\dots ,d$. Given a $\lambda$-connection of the form $$\nabla _\lambda =\lambda \partial _x+\left( {\begin{matrix} \varphi _0 &{} \;\;\varphi _+\\ \varphi _- &{} -\varphi _0\end{matrix}}\right) $$ it can be shown by an elementary calculation that $\nabla _\lambda$ can be brought to oper form $$\lambda \partial _x+\left( {\begin{matrix} 0 &{} q_\lambda \\ 1 &{} 0\end{matrix}}\right) $$ by means of a gauge transformation *h*,

3.11 $$\begin{aligned} \nabla _{\mathrm{\scriptscriptstyle Op}}=h^{-1}\cdot \nabla _\lambda \cdot h=\lambda \partial _y+\bigg (\,\begin{matrix} 0 &{} q_\lambda \\ 1 &{} 0\end{matrix}\bigg ), \end{aligned}$$

which is well-defined on a cover of *C* branched at the zeros $x_k$, $k=1,\dots ,d$, of $\varphi _-=\varphi _-(x)$. The resulting formula for the matrix element $q_\lambda$ is found to be of the form

3.12 $$\begin{aligned} q_\lambda (x)=\sum _{r=1}^n\left( \frac{a_r^2}{(x-z_r)^2}+\frac{H_r}{x-z_r}\right) +\lambda \sum _{k=1}^{d}\left( \frac{y_k}{x-x_k} -\frac{3\lambda }{4(x-x_k)^2}\right) . \end{aligned}$$

Assuming that $\nabla _\lambda$ is holomorphic on $C_{0,n}$, it follows from (3.11) that the monodromy of $\nabla _{\mathrm{\scriptscriptstyle Op}}$ around the points $x_k$ is proportional to the identity matrix and therefore trivial in ${\textrm{PSL}}(2,{{\mathbb {C}}})$. Singularities having this property are called apparent singularities. Having an apparent singularity at $x=x_k$ is equivalent to the fact that the parameters $(x_k,y_k)$ introduced in (3.12) satisfy the equations

3.13 $$\begin{aligned} \lambda ^2y_k^2+{q}_0^{(k)}=0, \quad k=1, \dots , d,\qquad q_\lambda (x)=\sum _{n=-2}(x-x_k)^n q_n^{(k)}. \end{aligned}$$

Taking into account the constraints (3.13) and the constraints from regularity at infinity it is not hard to see that for fixed $a_r$, $r=1,\dots ,n$, in (3.12) one gets a family of quadratic differentials $q_\lambda$ on *C* depending on $2(n-3)$ independent parameters.

Conversely, if the constraints (3.13) are satisfied, and if $d\le n-3$, there exists a unique gauge transformation *h* holomorphic on a double cover of $C_{0,n}{\setminus }\{x_1,\dots ,x_d\}$ with branch points only at $x_1,\dots ,x_d$ such that the connection $\nabla _\lambda$ defined from $$\nabla _{\mathrm{\scriptscriptstyle Op}}=\lambda \partial _x+\left( {\begin{matrix} 0 &{} q_\lambda \\ 1 &{} 0\end{matrix}}\right) $$ by means of (3.11) is holomorphic on $C_{0,n}$ with first order poles only at $x=z_r$. Indeed, by defining

3.14 $$\begin{aligned} \varphi _-(x)=c_0\frac{\prod _{k=1}^{d}(x-x_k)}{\prod _{r=1}^n(x-z_r)},\qquad \varphi _0(x)=\sum _{k=1}^d y_k\left( \prod _{r=1}^n\frac{x_k-z_r}{x-z_r}\right) \prod _{\begin{array}{c} l=1\\ l\ne k \end{array}}^d\frac{x-x_l}{x_k-x_l},\qquad \end{aligned}$$

and using these functions to build

3.15 $$\begin{aligned} h=\bigg (\begin{matrix} 1/\sqrt{\varphi _-} &{} 0\\ 0 &{} \sqrt{\varphi _-}\end{matrix}\bigg )\left( \begin{matrix} 1 &{} \alpha \\ 0 &{} 1\end{matrix}\right) , \quad \alpha (x)=\frac{\lambda }{2}\frac{\varphi _-'}{\varphi _-}-\varphi _0(x), \end{aligned}$$

we find that the connection $\nabla _\lambda$ is holomorphic on $C_{0,n}$.

Allowing quantum corrections containing apparent singularities therefore gives us a way to represent all the data characterising a gauge equivalence class of holomorphic connections in terms of meromorphic opers. The equivalence between flat ${{\mathfrak {s}}}{{\mathfrak {l}}}_2$-connections $\nabla _\lambda$ on $C_{0,n}$ and opers $\nabla _{\mathrm{\scriptscriptstyle Op}}$ observed above can be seen as a deformation of the Separation of Variables (SOV) for the classical Gaudin model [Sk, DM] with deformation parameter $\lambda$. Comparing with (3.3) we see that the positions $(x_1,\dots ,x_{d})$ of the apparent singularities are directly related to the divisor ${\mathbb {D}}$ characterising the line bundle ${{\mathcal {L}}}$ in the limit $\lambda \rightarrow 0$.

### Isomonodromic deformations

We are now going to observe that the deformation of the Higgs pairs $({{\mathcal {E}}},\varphi )$ into $\lambda$-connections leads to a natural deformation of the integrable flows of the Hitchin system, given by the isomonodromic deformation flows. It will turn out that this integrable structure controls how the free fermion partition function gets deformed when $\lambda$ is non-zero.

#### Riemann–Hilbert correspondence

The Riemann–Hilbert correspondence assigns holomorphic connections to representations $\rho :\pi _1(C)\rightarrow G$ of the fundamental group $\pi _1(C)$ in a group *G*, here taken to be $G={\textrm{SL}}(2,{{\mathbb {C}}})$. Considering curves *C* of genus 0 with a base point $x_0$ one may characterise the representations $\rho$ by the matrices $M_r$ representing closed curves $\gamma _r$ around the punctures $z_r$. We will consider the cases where the matrices $M_r$ are diagonalizable, $M_r=C_r^{-1}e^{2\pi {\textrm{i}}D_r}C_r^{}$, for a fixed choice of diagonal matrices $D_r$. The Riemann–Hilbert problem is to find a multivalued analytic matrix function $\Psi (x)$ on $C_{0,n}$ such that the monodromy along $\gamma _r$ is represented by

3.16 $$\begin{aligned} \Psi (\gamma _r.x)=\Psi (x)\cdot M_r, \end{aligned}$$

with $\Psi (\gamma _r.x)$ being the analytic continuation of $\Psi (x)$ along $\gamma _r$. The solution to this problem is unique up to left multiplication with single valued matrix functions. In order to fix this ambiguity we need to specify the singular behaviour of $\Psi (x)$ at $x=z_r$, leading to the following refined version of the Riemann–Hilbert problem:

*Find a matrix function* $\Psi (x)$ *such that (i)* $\Psi (x)$ *is a multivalued, analytic and invertible function on* $C_{0,n}$ *satisfying a normalisation condition, and (ii) there exist neighborhoods of* $z_k$, $k=1,\dots ,n$ *where* $\Psi (x)$ *can be represented as*

3.17 $$\begin{aligned} \Psi (x)\,=\,{\hat{Y}}^{(k)}(x)\cdot (x-z_k)^{D_k}\cdot C_k, \end{aligned}$$

*with* ${\hat{Y}}^{(k)}(x)$ *holomorphic and invertible at* $x=z_k$, $C_k\in G$, *and* $D_k$ *being diagonal matrices for* $k=1,\dots ,n$.

A standard choice of a normalisation condition is to require that $\Psi (x_0)=1$ at a fixed point $x_0\in C$. Other options are to fix the matrix ${\hat{Y}}^{(k)}(z_k)$ appearing in (3.17) for one particular value of *k*. If such a function $\Psi (x)$ exists, it is uniquely determined by the monodromy data ${\textbf{C}}=(C_1,\dots ,C_n)$ and the diagonal matrices ${\textbf{D}}=(D_1,\dots ,D_n)$. It is known that the solutions to the Riemann–Hilbert problem exist for generic representations $\rho :\pi _1(C_{0,n})\rightarrow G$.

#### Isomonodromic deformations

We shall now briefly indicate how the Riemann–Hilbert problem is related to the isomonodromic deformation problem. Given a solution $\Psi (x)=\Psi (x;\mu ,{\textbf{z}})$ to the Riemann–Hilbert problem we may define a connection *A*(*x*) as

3.18 $$\begin{aligned} A(x):=\,(\partial _x \Psi (x))\cdot (\Psi (x))^{-1}, \end{aligned}$$

It follows from (ii) that *A*(*x*) is a rational function of *x* which has the form

3.19 $$\begin{aligned} A(x)\,=\,\sum _{r=1}^{n}\frac{A_r(\mu ,{\textbf{z}})}{x-z_r}, \end{aligned}$$

${\textbf{z}}=(z_1,\dots ,z_n)$ being the positions of the punctures. A variation of ${\textbf{z}}$ for fixed monodromy data $\mu$ leads to a variation of the matrix residues $A_r$. It is not hard to show (see e.g. [BBT]) that the resulting variations are described by a nonlinear first order system of partial differential equations called the Schlesinger equations. The Schlesinger equations are the Hamiltonian flows defined by the Hamiltonians and Poisson structure

3.20 $$\begin{aligned} H_r=\sum _{s\ne r} \frac{\textrm{tr}(A_rA_s)}{z_r-z_s}, \qquad \big \{A(x)\,\begin{array}{c} \otimes \ \, \end{array}\,A(x')\big \}= \left[ \frac{{\mathcal {P}}}{x-x'},A(x)\otimes 1+1\otimes A(x')\right] \!,\qquad \end{aligned}$$

where ${\mathcal {P}}$ denotes the permutation matrix.

With the help of the equivalence between holomorphic connections and meromorphic opers one may describe the isomonodromic deformation flows as the flows describing isomonodromic deformations of the second order differential operator ${{\mathcal {D}}}_{q_\lambda }$. It is worth noting that

(i) the Hamiltonians $H_r$ generating the isomonodromic deformation flows are related to the residues $H_r$ in (3.12) by the gauge transformation from holomorphic connections to opers with apparent singularities,
(ii) the equations (3.13) are a system of linear equations for the residues $H_r$ in (3.12) which can be solved explicitly to get $H_r\equiv H_r({\textbf{x}},{\textbf{y}};\lambda )$, ${\textbf{x}}=(x_1,\dots ,x_{n-3})$, ${\textbf{y}}=(y_1,\dots ,y_{n-3})$,
(iii) the isomonodromic deformation equations can then be represented in Hamiltonian form as
  3.21 $$\begin{aligned} \frac{\partial x_k}{\partial z_r}=\frac{\partial H_r}{\partial y_k},\qquad \frac{\partial y_k}{\partial z_r}=-\frac{\partial H_r}{\partial x_k}, \end{aligned}$$
(iv) the coordinates $({\textbf{x}},{\textbf{y}})$ are Darboux coordinates for the Poisson structure (3.20), as equations (3.21) suggest.

The proofs of these statements can be found in [Ok, IKSY, DM]. In this form it becomes easy to see that the isomonodromic deformation flows turn into flows of the Hitchin integrable system for $\lambda \rightarrow 0$, with $({\textbf{x}},{\textbf{y}})$ being the variables in the SOV representation [DM]. One may recall, in particular, that the variables $x_k$ defining the divisor ${\mathbb {D}}$ are nothing but the zeros of $\varphi _-(x)$, and note that the functions $H_r({\textbf{x}},{\textbf{y}};\lambda )$ turn into the Hamiltonians of the Hitchin system for $\lambda \rightarrow 0$.

### Isomonodromic tau-function

The isomonodromic tau-function ${\mathcal {T}}(\mu ,{\textbf{z}})$ is then defined as the generating function for the Hamiltonians $H_r$,

3.22 $$\begin{aligned} H_r\,=\, \partial _{z_r}\log {\mathcal {T}}(\mu ,{\textbf{z}}). \end{aligned}$$

It can be shown that the integrability of (3.22) is a direct consequence of the Schlesinger equations. Equation (3.22) determines ${\mathcal {T}}(\mu ,{\textbf{z}})$ only up to addition of a function of the monodromy data. Having fixed this freedom by suitable supplementary conditions, one may use the Schlesinger equations to determine the dependence of ${\mathcal {T}}(\mu ,{\textbf{z}})$ on ${\textbf{z}}$ via (3.20) and (3.22).

We will see in the following that the free fermion partition functions we want to associate to the ${{\mathcal {D}}}$-modules representing the quantum curves can be identified with the isomonodromic tau functions coming from the Riemann–Hilbert problem characterising the relevant ${{\mathcal {D}}}$-modules.

## From Quantum Curves to Free Fermion Partition Functions

We are now going to explain how to define free fermion partition functions from the solutions of the differential equation defining the quantum curve. This construction generalises the deformed version of the Krichever construction used in [DHS]. The relation to the theory of infinite Grassmannians and of the Sato–Segal–Wilson tau-functions used in [DHS] is explained in Appendix A. The free fermion partition functions defined in this way turn out to be closely related to conformal blocks of the free fermion vertex operator algebra (VOA). The conformal Ward identities determininig the dependence of the free fermion partition functions with respect to the complex structure of *C* are equivalent to the equations defining the isomonodromic tau-functions. It will follow that a suitable choice of normalisation factors, which may still depend on the monodromy data characterising the equation of the quantum curve through the Riemann–Hilbert correspondence, allows us to relate the free fermion partition functions of our interest to isomonodromic tau-functions.

### From ${{\mathcal {D}}}$-modules to free fermion states

#### Free fermions

The free fermion super VOA is generated by fields $\psi _s(z)$, ${\bar{\psi }}_s(z)$, $s=1,\dots ,N$, The fields $\psi _s(z)$ will be arranged into a row vector $\psi (z)=(\psi _1(z),\dots ,\psi _N(z))$, while ${{\bar{\psi }}}(z)$ will be our notation for the column vector with components ${\bar{\psi }}_s(z)$. The modes of $\psi (z)$ and ${{\bar{\psi }}}(z)$, introduced as

4.1 $$\begin{aligned} \psi (z)=\sum _{n\in {{\mathbb {Z}}}}\psi _{n} z^{-n-1},\quad {{\bar{\psi }}}(z)=\sum _{n\in {{\mathbb {Z}}}} {{\bar{\psi }}}_{n} z^{-n}, \end{aligned}$$

are row and column vectors with components $\psi _{s,n}$ and ${\bar{\psi }}_{s,n}$, respectively, satisfying

4.2 $$\begin{aligned} \{\,\psi _{s,n},\,{{\bar{\psi }}}_{t,m}\,\}=\delta _{s,t}\delta _{n,-m},\qquad \{\,\psi _{s,n},\,\psi _{t,m}\,\}=0,\qquad \{\,{{\bar{\psi }}}_{s,n},\,{{\bar{\psi }}}_{t,m}\,\}=0. \end{aligned}$$

The Fock space ${{\mathcal {F}}}$ is a representation generated from a highest weight vector ${\mathfrak {f}}_{0}^{}$ satisfying

4.3 $$\begin{aligned} \psi _{s,n}\cdot {\mathfrak {f}}_{0}^{}=0,\quad n\ge 0,\qquad {{\bar{\psi }}}_{s,n}\cdot {\mathfrak {f}}_{0}^{}=0,\quad n>0. \end{aligned}$$

${{\mathcal {F}}}$ is generated from ${\mathfrak {f}}_{0}^{}$ by the action of the modes $\psi _{s,n}$, $n<0$, and ${{\bar{\psi }}}_{s,m}$, $m\le 0$.

We will also consider the conjugate representation ${{\mathcal {F}}}^*$, a *right* module generated from a highest weight vector ${\mathfrak {f}}_{0}^{*}$ satisfying

4.4 $$\begin{aligned} {\mathfrak {f}}_{0}^{*}\cdot \psi _{s,n}=0,\quad n< 0,\qquad {\mathfrak {f}}_{0}^{*}\cdot {{\bar{\psi }}}_{s,n}\,=0,\quad n\le 0. \end{aligned}$$

The Fock space ${{\mathcal {F}}}^*$ is generated from ${\mathfrak {f}}_{0}^{*}$ by the right action of the modes $\psi _{s,n}$, $n\ge 0$, and ${{\bar{\psi }}}_{s,m}$, $m> 0$. A natural bilinear form ${{\mathcal {F}}}^*\otimes {{\mathcal {F}}}\rightarrow {{\mathbb {C}}}$ is defined by the expectation value,

4.5 $$\begin{aligned} \langle \,{\mathfrak {f}}_0^*\cdot {\textsf{O}}_{{\mathfrak {f}}^*},\,{\textsf{O}}_{{\mathfrak {f}}}\cdot {\mathfrak {f}}_0\,\rangle = \Omega ({\textsf{O}}_{{\mathfrak {f}}^*}{\textsf{O}}_{{\mathfrak {f}}}\cdot {\mathfrak {f}}_0), \end{aligned}$$

where $$\Omega ({\mathfrak {f}})=c$$ if $${\mathfrak {f}}= c\,{\mathfrak {f}}_{0}^{}+\sum _{s=1}^N(\sum _{n<0} \psi _{s,n}{\mathfrak {f}}_{s,n} + \sum _{m\le 0}{\bar{\psi }}_{s,m}{\mathfrak {f}}_{s,m})$$.

#### Free fermion states from the Riemann–Hilbert correspondence

A simple and natural way to characterise a state ${\mathfrak {f}}\equiv {\mathfrak {f}}_G\in {{\mathcal {F}}}$ is through the matrix $G(x,y)\equiv G_{\mathfrak {f}}(x,y)$ of two-point functions having matrix elements

4.6 $$\begin{aligned} G_{\mathfrak {f}}(x,y)_{st}=\big \langle \,{\bar{\psi }}_s(x)\psi _t(y) \,\big \rangle _{{\mathfrak {f}}} \equiv \frac{\langle \,{\mathfrak {f}}_{0}^{*},\,{\bar{\psi }}_s(x)\psi _t(y)\,{\mathfrak {f}}\,\rangle }{\langle \, {\mathfrak {f}}_{0}^{*},\,{\mathfrak {f}}\,\rangle }. \end{aligned}$$

Indeed, given a function *G*(*x*, *y*) such that

4.7 $$\begin{aligned} G(x,y)=\frac{{1}}{x-y}+A(x,y), \end{aligned}$$

with *A*(*x*, *y*) having an expansion of the form

4.8 $$\begin{aligned} A(x,y)=\sum _{l\ge 0} y^{-l-1}\sum _{k>0}x^{-k}A_{kl}, \end{aligned}$$

there exists a state ${\mathfrak {f}}_G$, unique up to normalisation, such that its two-point function is given by *G*(*x*, *y*). States ${\mathfrak {f}}_G^{}$ having this property can be constructed as

4.9 $$\begin{aligned} {\mathfrak {f}}_G^{} =N_G^{}\exp \bigg (-\sum _{k>0}\sum _{l\ge 0} \psi _{-k}\cdot A_{kl}\cdot {\bar{\psi }}_{-l}\bigg ) {\mathfrak {f}}_{0}^{}, \end{aligned}$$

with matrices $A_{kl}$ defined by the expansion (4.8), and $N_G\in {{\mathbb {C}}}$ being a normalisation constant. This can be verified by a straightforward computation.

We will be mainly interested in two-point functions *G*(*x*, *y*) that have a multi-valued analytic continuation with respect to both *x* and *y* to the Riemann surfaces $C=C_{0,n}$ with given monodromies. The monodromies describing the analytic continuation in *x* are required to act on *G*(*x*, *y*) from the left, while the analytic continuation in *y* generates monodromies acting from the right. Consistency with having a pole at $x=y$ with residue being the identity matrix requires

4.10 $$\begin{aligned} G(x,\gamma _r.y)=G(x,y)\cdot M_r,\qquad G(\gamma _r.x,y)=M_r^{-1}\cdot G(x,y). \end{aligned}$$

This means that the family of functions $G_x(y):=G(x,y)$ is a solution to a generalisation of the Riemann–Hilbert problem formulated above where one allows a first order pole at $y=x$, and the family $G_y(x):=G(x,y)$ is a solution to a conjugate version of this Riemann–Hilbert problem. Uniqueness of the solution to the Riemann–Hilbert problem implies that *G*(*x*, *y*) must have the following form

4.11 $$\begin{aligned} G_{\Psi }(x,y)=\frac{(\Psi (x))^{-1}\Psi (y)}{x-y}, \end{aligned}$$

with $\Psi (y)$ being a solution to the Riemann–Hilbert problem formulated in Sect. 3.3.1.

The construction of the fermionic states ${\mathfrak {f}}_{G}$ described above therefore gives us a natural way to assign fermionic states ${\mathfrak {f}}_\Psi ^{}\equiv {\mathfrak {f}}_{G_\Psi }$ to solutions $\Psi$ of the Riemann–Hilbert problem.

### Free fermion conformal blocks from ${{\mathcal {D}}}$-modules

We are now offering a useful change of perspective by re-interpreting the fermionic states associated to ${{\mathcal {D}}}$-modules as free fermion conformal blocks. This will allow us to use methods and ideas from conformal field theory which will be useful for the computation of tau-functions. To this aim we will note that the states ${\mathfrak {f}}_\Psi ^{}\in {{\mathcal {F}}}$ constructed in Sect. 4.1.2 are characterised by a set of Ward identities defined from a solution $\Psi (x)$ of the RH problem. Given that conformal blocks can be defined as solutions to such Ward identities^8^ we are led to identify the states ${\mathfrak {f}}_\Psi ^{}\in {{\mathcal {F}}}$ as conformal blocks for the free fermion VOA.

Let us define the following infinite-dimensional spaces of multi-valued functions on ${C}_{0,n}$,

4.12 $$\begin{aligned} \begin{aligned}&{\bar{W}}_\Psi =\big \{\,{\bar{v}}(x)\cdot \Psi (x);\;\,{\bar{v}}(x) \in {\mathbb {C}}^N\otimes {\mathbb {C}}[{\mathbb {P}}^1\!{\setminus }\!\{\infty \}]\,\big \},\\&{{W}}_\Psi =\big \{\,\Psi ^{-1}(x)\cdot {v}(x);\;\,{v}(x)\in {\mathbb {C}}^N\otimes {\mathbb {C}}[{\mathbb {P}}^1\!{\setminus }\!\{\infty \}]\,\big \}, \end{aligned} \end{aligned}$$

where ${\bar{v}}$ and *v* are row and column vectors with *N* components, respectively, and ${\mathbb {C}}[{\mathbb {P}}^1\!{\setminus }\!\{\infty \}]$ is the space of meromorphic functions on ${\mathbb {P}}^1$ having poles at $\infty$ only. The elements of the space ${\bar{W}}_\Psi$ represent solutions of a generalisation of the RH problem from Sect. 3.3.1 where the condition of regularity at infinity has been dropped.

Let us next note that the vectors ${\mathfrak {f}}_\Psi ^{}$ defined in (4.9) can be equivalently characterised up to normalisation by the conditions

4.13 $$\begin{aligned} \psi [g]\cdot {\mathfrak {f}}_\Psi ^{}=0,\qquad {\bar{\psi }}[{\bar{f}}\,]\cdot {\mathfrak {f}}_\Psi ^{}=0, \end{aligned}$$

for all $g\in W_\Psi$, ${\bar{f}}\in {\bar{W}}_\Psi$, where the operators $\psi [{\bar{f}}\,]$ are constructed as

4.14 $$\begin{aligned} \psi [{g}]\,=\,\frac{1}{2\pi {\textrm{i}}}\int _{{{\mathcal {C}}}} dz\; \psi (z)\cdot {g}(z),\qquad {\bar{\psi }}[{\bar{f}}\,]\,=\,\frac{1}{2\pi {\textrm{i}}}\int _{{{\mathcal {C}}}} dz\; {\bar{f}}(z)\cdot {\bar{\psi }}(z), \end{aligned}$$

with ${{\mathcal {C}}}$ being a circle separating $\infty$ from $z_1,\dots ,z_n$.

Indeed, it can easily be shown that the vector ${\mathfrak {f}}_\Psi ^{}$ is defined uniquely up to normalisation by the identities (4.13). Let us note that the columns of ${\bar{G}}_l(x)$, $l\ge 0$, and the rows of the matrix-valued functions $G_k(y)$, $k>0$, defined through the expansions

4.15 $$\begin{aligned} \frac{(\Psi (x))^{-1}\Psi (y)}{x-y}=\left\{ \begin{aligned}&\sum _{l\ge 0} y^{-l-1} {\bar{G}}_l(x),\quad {\bar{G}}_l(x)= -x^l\,{\textbf{1}}+\sum _{k>0}x^{-k}A_{kl},\\&\sum _{k> 0} x^{-k} {G}_k(y),\quad {G}_k(y)= y^{k-1}\,{\textbf{1}}+\sum _{l\ge 0}y^{-l-1}A_{kl}. \end{aligned}\right. \end{aligned}$$

generate bases for the spaces ${\bar{W}}_\Psi$ and ${W}_\Psi$ associated to $\Psi (x)$, respectively. The conditions (4.13) are equivalent to the validity of

4.16 $$\begin{aligned} {\bar{\psi }}_k^{}\,{\mathfrak {f}}_\Psi ^{}=-\sum _{l\ge 0}(A_{kl}\cdot {\bar{\psi }}_{-l}^{})\,{\mathfrak {f}}_\Psi ^{},\qquad {\psi }_l^{}\,{\mathfrak {f}}_\Psi ^{}=\sum _{k> 0}({\psi }_{-k}^{}\cdot A_{kl})\,{\mathfrak {f}}_\Psi ^{}, \end{aligned}$$

for all $k>0$ and all $l\ge 0$. The identities (4.16) can be used to calculate the values of $\langle {\mathfrak {v}},{\mathfrak {f}}_\Psi ^{}\rangle _{{\mathcal {F}}}$ for ${\mathfrak {f}}_\Psi ^{}\in {{\mathcal {F}}}$ satisfying (4.13) and arbitrary ${\mathfrak {v}}\in {{\mathcal {F}}}$ in terms of $\langle {\mathfrak {f}}_{0}^{},{\mathfrak {f}}_\Psi ^{}\rangle _{{\mathcal {F}}}$. This implies that the solution to the conditions (4.13) is unique up to normalisation. It is not hard to check that the vector ${\mathfrak {f}}_\Psi ^{}$ defined using (4.15) and (4.9) indeed satisfies the identities (4.16).

The definition of ${\mathfrak {f}}_\Psi ^{}$ through the identities (4.13) is analogous to the definition of Virasoro conformal blocks through the conformal Ward identities. The uniqueness of ${\mathfrak {f}}_\Psi ^{}$ implies that the space of conformal blocks for the free fermionic VOA is one-dimensional.

### Chiral partition functions as isomonodromic tau-functions

Out of a representation of the free fermion VOA one may define a representation of the Virasoro algebra by introducing the energy-momentum tensor as

4.17 $$\begin{aligned} T(z)=\frac{1}{2}\lim _{w\rightarrow 0}\sum _{s=1}^N\bigg (\partial _z\psi _s(w){\bar{\psi }}_s(z)+\partial _z{\bar{\psi }}_s(w)\psi _s(z)+\frac{1}{(w-z)^2}\bigg ). \end{aligned}$$

Conformal blocks for the free fermion VOA represent conformal blocks for the Virasoro algebra defined via (4.17). On the space of conformal blocks of the Virasoro algebra there is a canonical connection [FS] allowing us to represent the variations of a conformal block induced by variations of the complex structure of the underlying Riemann surface $C_{0,n}$ in the form^9^

4.18 $$\begin{aligned} \partial _{z_r}{\mathfrak {f}}_\Psi ^{}={\textsf{H}}_r\, {\mathfrak {f}}_\Psi ^{}, \end{aligned}$$

with ${\textsf{H}}_r$ being suitable linear combinations of the modes of *T*(*z*). This connection preserves the one-dimensional space of free fermion conformal blocks due to the fact that the adjoint action of the Virasoro algebra acts geometrically on the free fermions, transforming them as half-differentials.

The operators ${\textsf{H}}_r$ generate a commutative subalgebra of the Virasoro algebra, embedded into the Lie algebra generated by fermion bilinears via (4.17). Keeping in mind the fact that only the normalisation of ${\mathfrak {f}}_\Psi ^{}$ was left undetermined by (4.13) one sees that the equations (4.18) together with (4.13) can be used to determine ${\mathfrak {f}}_{\Psi }({\textbf{z}})$ unambiguously in terms of ${\mathfrak {f}}_{\Psi }({\textbf{z}}_0)$ for any given path connecting ${\textbf{z}}$ and ${\textbf{z}}_0$ in ${{\mathcal {M}}}_{0,n}$, the moduli space of complex structures on $C_{0,n}$. Using only the Ward identities one can show that^10^

4.19 $$\begin{aligned} \partial _{z_r}\log \langle \,{\mathfrak {f}}_{0}^{},\,{\mathfrak {f}}_\Psi ^{}({\textbf{z}})\,\rangle = \frac{\langle \,{\mathfrak {f}}_{0}^{},{{\textsf{H}}}_r\,{\mathfrak {f}}_\Psi ^{}({\textbf{z}})\,\rangle }{\langle \,{\mathfrak {f}}_{0}^{},\,{\mathfrak {f}}_\Psi ^{}({\textbf{z}})\,\rangle } = H_r(\mu ,{\textbf{z}}), \end{aligned}$$

with $H_r$ being the isomonodromic deformation Hamiltonians defined in (3.20). This means that the isomonodromic tau-function coincides up to a function $N(\mu )$ of the monodromy data with

4.20 $$\begin{aligned} {\mathcal {Z}}_{\textrm{ff}}(\mu ,{\textbf{z}})=N(\mu ){{\mathcal {T}}}(\mu ,{\textbf{z}}),\qquad {\mathcal {Z}}_{\textrm{ff}}(\mu ,{\textbf{z}}):= \langle \,{\mathfrak {f}}_{0}^{},\,{\mathfrak {f}}_{\Psi }^{}({\textbf{z}})\rangle , \end{aligned}$$

relating the isomonodromic tau-functions to free fermion conformal blocks.

#### Remark 1

Starting from a Lagrangian description of the free fermions on a Riemann surface *C* one would naturally arrive at a description of the free fermion partition functions as determinants of Cauchy–Riemann-operators on *C*. Such determinants have been studied for $C=C_{0,n}$ in [Pa] where it was shown that they are related to the isomonodromic tau-functions. This offers an alternative approach to the relation between free fermion partition functions and isomonodromic tau-functions expressed in (4.20).

A solution to the Riemann Hilbert problem has first been constructed using fermionic twist fields in [SMJ], and the relation to conformal field theory was previously discussed in [Mo].

### Issues to be addressed

Two points should be noted at this stage: First, let us note that the Riemann–Hilbert correspondence relates the moduli space ${{\mathcal {M}}}_{\textrm{flat}}(C_{0,n})$ of flat connections $\partial _y-A(y)$ on $C_{0,n}$ to the character variety ${{\mathcal {M}}}_{\textrm{ch}}(C_{0,n})=\textrm{Hom}(\pi _1(C_{0,n}),\textrm{SL}(2,{{\mathbb {C}}}))/\textrm{SL}(2,{{\mathbb {C}}})$. The definition above therefore defines the tau-function as a function of two types of data: The variables ${\textbf{z}}$ specifying the complex structure of *C*, and the monodromy data *M*, represented by the matrices $M_r$ appearing in the Riemann–Hilbert problem. Picking a parameterisation $M_r=M_r(\mu )$, $\mu =(\mu _1,\dots ,\mu _{2n-6})$, of the monodromy data $M_r$ is equivalent to introducing coordinates $\mu$ for the character variety. Doing this will allow us to represent the tau-functions as actual functions ${\mathcal {T}}(\mu ,{\textbf{z}})$ depending on two types of variables. The identification of the tau-function ${\mathcal {T}}(\mu ,{\textbf{z}})$ with the free fermion partition function $Z_{\textrm{ff}}(\xi ,t;\lambda )$ must therefore involve a map between the variables $(t,\xi )$ and the geometric data $(\mu ,{\textbf{z}})$ that needs to be determined.

Second, the definition above defines the tau-function up to multiplication with functions of the monodromy data which do not depend on ${\textbf{z}}$. For the time being we will call a tau-function *any* function ${\mathcal {T}}(\mu ,{\textbf{z}})$ satisfying $H_r=\partial _{z_r}\log {\mathcal {T}}(\mu ,{\textbf{z}})$, $r=1,\dots ,n-3$. We will later find natural ways to fix this ambiguity. Remarkably it will turn out that the choice of coordinates $\mu$ for ${{\mathcal {M}}}_{\textrm{ch}}(C_{0,n})$ will determine natural ways for fixing the normalisation of ${\mathcal {Z}}_{\textrm{ff}}(\mu ,{\textbf{z}})$.

## Factorising the Tau-Functions

The definition of the free fermion partition functions given in the previous section, elegant as it may be, is not immediately useful for computations. Recently it has been shown in [GIL, ILT] how to compute the series expansions for the isomonodromic tau-functions ${{\mathcal {T}}}(\mu ,{\textbf{z}})$ in cross-ratios of the positions $z_r$ explicitly. This result has been re-derived in [GL16] by a different method which can be seen as a special case of the general relations between Riemann–Hilbert factorisation problems and tau-functions discussed in [CGL].

In this section we are going to explain how the existence of the combinatorial expansions found in the references above is naturally explained from the theory of free chiral fermions. The factorisation over a complete set of intermediate states will lead to expressions which in the case $C=C_{0,4}$ take the schematic form

5.1 $$\begin{aligned} {{\mathcal {T}}}(\sigma ,\kappa ;z)=\sum _{n\in {{\mathbb {Z}}}}e^{in\kappa }{{\mathcal {T}}}_n(\sigma ;z). \end{aligned}$$

This will allow us to determine the precise relation between the variables $\sigma ,\kappa$ in (5.1) and certain coordinates for the moduli space ${{\mathcal {M}}}_{\textrm{flat}}(C_{0,4})$ of flat *SL*(2)-connections on $C_{0,4}$, addressing one of the main issues formulated at the end of Sect. 4.

### Coordinates from factorisation of Riemann–Hilbert problems

Let us first discuss how the factorisation of Riemann–Hilbert problems leads to the definition of coordinates for the space of monodromy data. Within this subsection we will specialise to the case $N=2$.

#### Fenchel-Nielsen type coordinates

Useful sets of coordinates for ${{\mathcal {M}}}_{\textrm{ch}}(C_{g,n})$ are given by the trace functions $L_{\gamma }:=\textrm{tr}\rho (\gamma )$ associated to simple closed curves $\gamma$ on $C_{g,n}$ [Go]. Conjugacy classes of irreducible representations of $\pi _1(C_{0,4})$ are uniquely specified by seven conjugation invariants

5.2a $$\begin{aligned}&L_k={\text {Tr}} M_k=2\cos 2\pi \theta _k,\qquad k=1,\ldots ,4, \end{aligned}$$

5.2b $$\begin{aligned}&L_s={\text {Tr}} M_1 M_2,\qquad L_t={\text {Tr}} M_1 M_3,\qquad L_u={\text {Tr}} M_2 M_3, \end{aligned}$$

generating the algebra of invariant polynomial functions on ${{\mathcal {M}}}_{\textrm{char}}(C_{0,4})$. These trace functions satisfy the quartic equation

5.3 $$\begin{aligned}&L_1L_2L_3L_4+L_sL_tL_u+L_s^2+L_t^2+L_u^2+L_1^2+L_2^2+L_3^2+L_4^2\nonumber \\&\quad =\left( L_1L_2+L_3L_4\right) L_s+\left( L_1L_3+L_2L_4\right) L_t +\left( L_2L_3+L_1L_4\right) L_u+4. \end{aligned}$$

For fixed choices of $\theta _1,\ldots ,\theta _4$ in (5.2a) one may use equation (5.3) to describe the character variety as a cubic surface in ${{\mathbb {C}}}^3$. This surface admits a parameterisation in terms of coordinates $(\sigma ,\tau )$ of the form

5.4 $$\begin{aligned} L_s\,=\,2\cos 2\pi \sigma ,\qquad \begin{aligned}&(2\sin (2\pi \sigma ))^2\,L_t\,=\, C_{t}^+(\sigma )\,e^{i\kappa }+C_t^0(\sigma )+C_{t}^-(\sigma )\,e^{-i\kappa },\\&(2\sin (2\pi \sigma ))^2\,L_u\,=\, C_{}^+(\sigma )\,e^{i\kappa }+C_u^0(\sigma )+C_{}^-(\sigma )\,e^{-i\kappa }, \end{aligned} \end{aligned}$$

where $$C_t^{\pm }(\sigma )=-C^{\pm }(\sigma )e^{\pm 2\pi \,{\textrm{i}}\,\sigma }$$,

5.5 $$\begin{aligned} \begin{aligned} C_t^0(\sigma )&=L_s(L_2L_3+L_1L_4)-{2 }(L_1L_3+L_2L_4)\\ C_u^0(\sigma )&=L_s(L_1L_3+L_2L_4)-{2 }(L_2L_3+L_1L_4). \end{aligned} \end{aligned}$$

Equation (5.3) only constrains the product $C_{}^+(\sigma )C_{}^-(\sigma )$, leaving the freedom to trade a redefinition of $\kappa$ in (5.4) for a redefinition of $C_{}^+(\sigma )$ and $C_{}^-(\sigma )$ which leaves $C_{}^+(\sigma )C_{}^-(\sigma )$ unchanged. We will in the rest of this subsection discuss natural ways to fix this ambiguity. The coordinates defined in this way will be called coordinates of Fenchel-Nielsen type.

#### Factorising Riemann–Hilbert problems

Let us assume $|z|<1$. We may represent the surfaces $C_{0,4}={\mathbb {P}}^1{\setminus }\{0,z,1,\infty \}$ by gluing two three-punctured spheres $C^{\textrm{in}}$ and $C^{\textrm{out}}$. Let us represent both $C^{\textrm{in}}$ and $C^{\textrm{out}}$ as ${\mathbb {P}}^1{\setminus }\{0,1,\infty \}$, and let $A^{\textrm{in}}=\{x\in C^{\textrm{in}};|1|<|x|<|z|^{-1}\}$ and $A^{\textrm{out}}=\{x\in C^{\textrm{out}};|z|<|x|<1\}$ be annuli in $C^{\textrm{in}}$ and $C^{\textrm{out}}$, respectively. By identifying points *x* in $A^{\textrm{in}}$ with points $x'$ in $A^{\textrm{out}}$ iff $x'=zx$ one recovers the Riemann surface $C_{0,4}$ from $C^{\textrm{in}}$ and $C^{\textrm{out}}$.

Having represented the Riemann surface $C_{0,4}$ by means of the gluing construction there is an obvious way to define Riemann–Hilbert problems for $C^{\textrm{in}}$ and $C^{\textrm{out}}$ using the matrices $M_1,M_{2}$ and $M_{3},M_4$, respectively. A solution $\Psi (x)$ to the Riemann–Hilbert problem on $C_{0,4}$ allows us to define solutions $\Psi ^{\textrm{in}}(x)$ and $\Psi ^{\textrm{out}}(x)$ to the corresponding Riemann–Hilbert problems on the open surfaces $D^{\textrm{in}}=\{x\in {{\mathbb {C}}};|x|<|z|^{-1}\}$ and $D^{\textrm{out}}=\{x\in {\mathbb {P}}^1;|x|>|z|\}$ in an obvious way, setting $\Psi ^{\textrm{out}}(x)=\Psi (x)T^{\textrm{in}}$ on $D^{\textrm{out}}$ and $\Psi ^{\textrm{in}}(x)=\Psi (zx)T^{\textrm{out}}$ on $D^{\textrm{in}}$, with $T^{\textrm{in}}, T^{\textrm{out}} \in {\textrm{SL}}(2,{{\mathbb {C}}})$ being fixed matrices describing a possible change of normalisation condition in the definition of the Riemann–Hilbert problems on $C^{\textrm{in}}$ and $C^{\textrm{out}}$. By choosing $T^{\textrm{in}}$, $T^{\textrm{out}}$ appropriately we can get functions $\Psi ^{\textrm{in}}(x)$ and $\Psi ^{\textrm{out}}(x)$ both having diagonal monodromy along the boundary circles of $D^{\textrm{in}}$ and $D^{\textrm{out}}$, respectively. The matrices $T^{\textrm{in}}, T^{\textrm{out}}$ which ensure this condition can only differ by a diagonal matrix, leading to a relation of the form $\Psi ^{\textrm{in}}(x)=\Psi ^{\textrm{out}}(zx)T,$ for $x\in A$.

Coordinates for the moduli space of flat connections ${{\mathcal {M}}}_{\textrm{flat}}(C_{0,4})$ can then be obtained by choosing a parameterisation for the two pairs of matrices $(M_1,M_{2})$ and $(M_3,M_4)$, and using the parameter $\kappa$ for the family of matrices $T_\kappa ={\textrm{diag}}(e^{{\textrm{i}}\kappa /2},e^{-{\textrm{i}}\kappa /2})$ as a complementary coordinate for ${{\mathcal {M}}}_{\textrm{flat}}(C_{0,4})$. An equivalent representation can be obtained by trading a nontrivial choice of the matrix *T* for an overall conjugation of $M_{1},M_2$ by *T*. It will be convenient to consider $\Psi ^{\textrm{in}}_{z,\kappa }(x):=\Psi ^{\textrm{in}}(x/z)T^{-1}$ instead of $\Psi ^{\textrm{in}}(x)$, which is related to $\Psi ^{\textrm{out}}(x)$ simply as $\Psi ^{\textrm{in}}_{z,\kappa }(x)=\Psi ^{\textrm{out}}(x)$ for $x\in A$.

#### Coordinates from the gluing construction

Representing $C=C_{0,4}$ by the gluing construction as described in Sect. 5.1.2 one needs the solutions of the Riemann–Hilbert problem for $C^{\textrm{in}}\simeq C_{0,3}$ and $C^{\textrm{out}}\simeq C_{0,3}$. It is a classical result that the solutions to the Riemann–Hilbert problem on $C_{0,3}$ can be expressed through the hypergeometric function. We may, in particular, choose $\Psi ^{\textrm{out}}$ as $$\Psi ^{\textrm{out}}(x)=\big ({\begin{matrix} \chi '_+ &{} \chi '_- \\ \chi _+ &{} \chi _- \end{matrix}}\big )$$, with

5.6 $$\begin{aligned} \begin{aligned} \chi _{\epsilon }(x)=\nu _\epsilon ^{\textrm{out}}\, x^{\frac{1}{2}+\epsilon (\sigma -\frac{1}{2})}(1-x)^{\frac{1}{2}+\epsilon \theta _3}F(A_\epsilon ,B_\epsilon ,C_\epsilon ;x), \end{aligned} \end{aligned}$$

for $\epsilon =\pm 1$, where $\nu _\epsilon ^{\textrm{out}}$ are normalisation factors to be specified later, *F*(*A*, *B*, *C*; *x*) is the Gauss hypergeometric function and

5.7 $$\begin{aligned} \begin{aligned} A_+=A, \quad A_-=1-A,\\ B_+=B, \quad B_-=1-B,\\ \end{aligned}\qquad \begin{aligned}&C_+=C,\\ {}&C_-=2-C, \end{aligned} \qquad \begin{aligned} A&=\theta _3+\theta _4+\sigma ,\\ B&=\theta _3-\theta _4+\sigma , \end{aligned}\qquad C=2\sigma .\qquad \end{aligned}$$

$\Psi ^{\textrm{in}}$, on the other hand, may be chosen as $$ \Psi ^{\textrm{in}}=\big ({\begin{matrix} \xi '_+ &{} \xi '_- \\ \xi _+ &{} \xi _- \end{matrix}}\big )$$, where $\xi _{\epsilon }(x)$ are obtained from $\chi _\epsilon (x)$ by the replacements $x\rightarrow x^{-1}$, $\theta _4\rightarrow \theta _1$, $\theta _3\rightarrow \theta _2$ and $\epsilon \rightarrow -\epsilon$.

The well-known formulae for the monodromies of the hypergeometric function then yield, in particular, formulae for the monodromy $M_3^{\textrm{out}}$ of $\Psi ^{\textrm{out}}(x)$ around $z_3=1$ of the form

5.8 $$\begin{aligned} M_3^{\textrm{out}}=\left( \begin{matrix} *&{} \mu _3^+ \\ \mu _3^- &{} *\end{matrix}\right) , \quad \mu _3^\epsilon =-\epsilon \,\left( \frac{\nu _+^{\mathrm{\scriptscriptstyle out}}}{\nu _-^{\mathrm{\scriptscriptstyle out}}}\right) ^{-\epsilon }\! \frac{2\pi {\textrm{i}}\,\Gamma (C_\epsilon )\Gamma (C_\epsilon -1)}{\Gamma (A_\epsilon )\Gamma (B_{\epsilon })\Gamma (C_\epsilon -A_\epsilon )\Gamma (C_\epsilon -B_{\epsilon })}.\qquad \end{aligned}$$

A similar formula gives the monodromy $M_2^{\textrm{in}}$ of $\Psi ^{\textrm{in}}(x)$ around 1. Keeping in mind the set-up introduced in Sect. 5.1.2 it is easy to see that ${\textrm{tr}}(M_2M_3)$ gets represented as

5.9 $$\begin{aligned} {\textrm{tr}}(M_2M_3)={\textrm{tr}}(T^{-1}M_2^{\textrm{in}}TM_3^{\textrm{out}})= e^{{\textrm{i}}\kappa }\mu _2^-\mu _3^++e^{-{\textrm{i}}\kappa }\mu _2^+\mu _3^-+N_0, \end{aligned}$$

where $N_0$ is $\kappa$-independent, and $T={\textrm{diag}}(e^{{\textrm{i}}\kappa /2},e^{-{\textrm{i}}\kappa /2})$. The parameters $(\sigma ,\kappa )$ introduced in this way represent coordinates for ${{\mathcal {M}}}_{\textrm{flat}}(C_{0,4})$ of Fenchel-Nielsen type. From equations (5.8) and (5.9) it is easy to see, in particular, that the definition of the coordinate $\kappa$ is directly linked to the choice of normalisation factors $\nu _\pm ^{\textrm{out}}$, $\nu _\pm ^{\textrm{in}}$ in the definition of $\Psi ^{\textrm{out}}$, $\Psi ^{\textrm{in}}$. It is furthermore natural to require that the determinants of $\Psi ^{\textrm{out}}(x)$ and $\Psi ^{\textrm{in}}(x)$ are equal to 1, fixing $\nu _+^{\textrm{out}}\nu _-^{\textrm{out}}$ and $\nu _+^{\textrm{in}}\nu _-^{\textrm{in}}$ to be equal to $\frac{1}{1-2\sigma }$, and leaving us with one undetermined normalisation constant.

Two choices appear to be particularly natural from this point of view. One may, on the one hand, choose $\nu _+^{\textrm{out}}=1$, $\nu _-^{\textrm{in}}=1$ in order to ensure that the coefficients appearing in the series expansions of $\Psi ^{\textrm{in}}(x)$ and $\Psi ^{\textrm{out}}(x)$ are rational functions of $\sigma$, $\theta _i$, $i=1,\dots ,4$. In that case we easily see that $C_{}^{\pm }(\sigma )=C_{\textrm{r}}^{\pm }(\sigma )$, with

5.10 $$\begin{aligned} C_{\textrm{r}}^{\pm }(\sigma )&= \frac{(2\pi )^2\,\Gamma (1\pm (2\sigma -1))^4}{ \prod _{s,s'=\pm 1}\Gamma \big (\frac{1}{2}\pm \big (\sigma -\frac{1}{2}\big )+s\theta _1+s' \theta _2\big )\Gamma \big (\frac{1}{2}\pm \big (\sigma -\frac{1}{2}\big )+s\theta _3+s' \theta _4\big )}. \end{aligned}$$

The normalisation factors $\nu _\pm ^{\textrm{out}}$ can alternatively be chosen such that $\mu _3^+=1$, which gives

5.11 $$\begin{aligned} (2\sin (2\pi \sigma ))^2\mu _3^-=-\prod _{s,s'=\pm 1} \,2\sin \pi (\sigma +s\theta _1+s' \theta _2). \end{aligned}$$

Adopting an analogous choice for $\nu _\pm ^{\textrm{in}}$ leads to $C_{}^+(\sigma ) = 1$ and

5.12 $$\begin{aligned} (2\sin (2\pi \sigma ))^4C_{}^{-}(\sigma )&=\prod _{s,s'=\pm 1} \,2\sin \pi (\sigma +s\theta _1+s' \theta _2)\,2\sin \pi (\sigma +s\theta _3+s' \theta _4)\nonumber \\&= (L_s^2+L_1^2+L_2^2-L_sL_1L_2-4)(L_s^2+L_3^2+L_4^2-L_sL_3L_3-4). \end{aligned}$$

It is worth noting that $C_{}^{\pm }(\sigma )$ are rational in $L_s$ in this parameterisation.

### Factorisation of free fermion conformal blocks

We had previously observed that the free fermion state ${\mathfrak {f}}_\Psi$ associated with the solution $\Psi$ of the Riemann–Hilbert problem on *C* defines a conformal block of the free fermion vertex algebra on *C*. A standard construction in conformal field theory allows us to represent conformal blocks on Riemann surfaces *C* obtained by gluing two surfaces $C^{\textrm{in}}$ and $C^{\textrm{out}}$ in terms of the conformal blocks associated to $C^{\textrm{in}}$ and $C^{\textrm{out}}$, respectively. Adapting this construction to our case will allow us to represent the free fermion partition functions as overlaps of the form

5.13 $$\begin{aligned} {\mathcal {Z}}_{\textrm{ff}}(\mu ,{z})= {\big \langle \,{\mathfrak {f}}_{\textrm{out}}^{*},\, {\mathfrak {f}}_{\textrm{in}}^{}\,\big \rangle _{{{\mathcal {F}}}}^{}}, \end{aligned}$$

where ${\mathfrak {f}}_{\textrm{out}}$, ${\mathfrak {f}}_{\textrm{in}}$ are states in the free fermion Fock space defined by factorising the RH problem along a contour $\gamma$ separating *C* into two open surfaces $C^{\textrm{out}}$ and $C^{\textrm{in}}$ as described in Sect. 5.1.2. The representation (5.13) for ${\mathcal {Z}}_{\textrm{ff}}(\mu ,{z})$ can be used to calculate the free fermion partition functions more explicitly.

#### Twisted representations

As a further preparation we will need to generalise the construction from Sect. 4.2 a bit. We will need twisted representations ${{\mathcal {F}}}_\sigma$ of the free fermion algebra labelled by a tuple $\sigma =(\sigma _1,\dots ,\sigma _N)\in {{\mathbb {C}}}^N$ where the fermions have non-trivial monodromy around $x=0$,

5.14 $$\begin{aligned} \psi _t(x)=\sum _{n\in {{\mathbb {Z}}}}\psi _{t,n} x^{-n-1+\sigma _t},\quad {{\bar{\psi }}}_s(x)=\sum _{n\in {{\mathbb {Z}}}} {{\bar{\psi }}}_{s,n} x^{-n-\sigma _s}, \end{aligned}$$

with $s,t=1,\dots ,N$. The twist fields describing such representations can be conveniently described by means of bosonisation. To this aim let us introduce *N* free bosonic fields,

5.15 $$\begin{aligned} \phi _s(x)={\textsf{q}}_s+{\textsf{p}}_s \log x+{\textrm{i}}\sum _{n\ne 0}\frac{1}{n}a_{s,n}x^{-n}, \end{aligned}$$

$s=1,\dots ,N$, having modes satisfying the commutation relations

5.16 $$\begin{aligned}{}[{\textsf{q}}_r,{\textsf{p}}_s]=\frac{{\textrm{i}}}{2}\delta _{r,s},\qquad [a_{r,m},a_{s,n}]=\frac{m}{2}\delta _{r,s}\delta _{n,-m}. \end{aligned}$$

We will consider Fock space representation ${{\mathcal {V}}}_{{\textbf{p}}}$ labelled by a tuple ${\textbf{p}}=(p_1,\dots ,p_N)$ generated from vectors ${v}_{{\textbf{p}}}$ satisfying

5.17 $$\begin{aligned} a_{n,s}\,{v}_{{\textbf{p}}}=0,\quad n>0, \qquad {\textsf{p}}_s \,{v}_{{\textbf{p}}}=p_s\, {v}_{{\textbf{p}}},\qquad e^{2{\textrm{i}}\delta {\textsf{q}}_s}{v}_{{\textbf{p}}}={v}_{{\textbf{p}}-\delta {\textbf{e}}_s}, \end{aligned}$$

for all $s=1,\dots ,N$, with ${\textbf{e}}_s$ being the unit vector having 1 at the *s*-th component, and $\delta \in {{\mathbb {R}}}$.

The direct sum of Fock spaces

5.18 $$\begin{aligned} {{\mathcal {F}}}_{\sigma }=\bigoplus _{{\textbf{n}}\in \frac{1}{2}{{\mathbb {Z}}}^{N}}{{\mathcal {V}}}_{\sigma +{\textbf{n}}}, \end{aligned}$$

is a representation of the free fermion VOA generated by the fields

5.19 $$\begin{aligned} \psi _s(x)=:e^{{\textrm{i}}\phi _s(x)}:,\qquad {\bar{\psi }}_s(x)=:e^{-{\textrm{i}}\phi _s(x)}:, \end{aligned}$$

from the vector ${\mathfrak {f}}_{\sigma }\equiv {v}_{\mathbf {\sigma }}$ satisfying the usual highest weight conditions. As before we may introduce a conjugate right module ${{\mathcal {F}}}_{\sigma }^*$. The spaces ${{\mathcal {F}}}_{\sigma }^*$ and ${{\mathcal {F}}}_\sigma$ are naturally paired by the bilinear form $\langle .,.\rangle _{{{\mathcal {F}}}_\sigma }^{}:{{\mathcal {F}}}_{\sigma }^*\otimes {{\mathcal {F}}}_\sigma \rightarrow {{\mathbb {C}}}$ defined in the same way as previously done for $\sigma =0$.

#### Representing conformal blocks within twisted representations

The construction of free fermion states corresponding to the solutions of the Riemann–Hilbert problem described in Section (4.1.2) can now easily be generalised to the cases where one of the points at which $\Psi (x)$ can be singular is equal to 0 or $\infty$. We will look for a state ${\mathfrak {f}}_{\Psi ,\sigma }^{}\in {{\mathcal {F}}}$ characterised through the matrix $G_\Psi (x,y)$ of two-point functions with matrix elements

5.20 $$\begin{aligned} G_\Psi (x,y)_{st}=\big \langle \,{\bar{\psi }}_s(x)\psi _t(y) \,\big \rangle _{\Psi } \equiv \frac{\langle \,{\mathfrak {f}}_{\sigma }^{},\,{\bar{\psi }}_s(x)\psi _t(y)\,{\mathfrak {f}}_{\Psi ,\sigma }^{}\,\rangle _{{{\mathcal {F}}}_\sigma }^{}}{\langle \, {\mathfrak {f}}_{\sigma }^{},\,{\mathfrak {f}}_{\Psi ,\sigma }^{}\,\rangle _{{{\mathcal {F}}}_\sigma }^{}}. \end{aligned}$$

However, in order to apply (4.9) and (4.8) we now need to use a modified form of the relation between the two-point function and the function *A*(*x*, *y*), taking into account that $\Psi (x)= \Phi (1/x)x^D$ near $x=\infty$, with *D* being the the diagonal matrix $D={\textrm{diag}}(\sigma _1,\dots ,\sigma _N)$, and $\Phi (x)$ regular at $x=0$. It follows that *A*(*x*, *y*) can be introduced via

5.21 $$\begin{aligned} G_{\Psi }(x,y)=\frac{(\Psi (x))^{-1}\Psi (y)}{x-y}=x^{-D}\left( \frac{{1}}{x-y}+A(x,y)\right) y^{D}, \end{aligned}$$

In a similar way one may define a state ${\mathfrak {f}}_{\Psi ,\sigma }^*\in {{\mathcal {F}}}_\sigma ^*$ such that

5.22 $$\begin{aligned} G_\Psi ^{}(x,y)_{st}=\big \langle \,{\bar{\psi }}_s(x)\psi _t(y) \,\big \rangle _{\Psi }^{} \equiv \frac{\langle \,{\mathfrak {f}}_{\Psi ,\sigma }^{*},\,{\bar{\psi }}_s(x)\psi _t(y)\,{\mathfrak {f}}_\sigma ^{}\,\rangle _{{{\mathcal {F}}}_\sigma }^{}}{\langle \, {\mathfrak {f}}_{\Psi ,\sigma }^{*},\,{\mathfrak {f}}_\sigma ^{}\,\rangle _{{{\mathcal {F}}}_\sigma }^{}}. \end{aligned}$$

The states ${\mathfrak {f}}_{\Psi ,\sigma }^{}$ and ${\mathfrak {f}}_{\Psi ,\sigma }^*$ are as before defined uniquely up to normalisation.

#### Factorisation of free fermion conformal blocks

Using these constructions, and referring back to the factorisation of the Riemann–Hilbert problem described in Sect. 5.1.2, we can now associate a state ${\mathfrak {f}}_{\textrm{in}}^{}\equiv {\mathfrak {f}}_{\textrm{in}}^{}(z,\kappa )\in {{\mathcal {F}}}_{\sigma }$ to $\Psi ^{\textrm{in}}_{z,\kappa }$, and a state ${\mathfrak {f}}_{\textrm{out}}^*\in {{\mathcal {F}}}_{\sigma }^*$ to $\Psi ^{\textrm{out}}$. Using the variable *z* as coordinate for ${{\mathcal {M}}}_{0,4}$ in the case $C=C_{0,4}$ one may, on the other hand, use (4.18) to define the family of states ${\mathfrak {f}}_{\Psi }^{}({z})$ up to a *z*-independent normalisation factor. We claim that ${\mathfrak {f}}_{\Psi }^{}({z})$ can be normalised in such a way that we have

5.23 $$\begin{aligned} {\mathcal {Z}}_{\textrm{ff}}(\mu ,{\textbf{z}})= \big \langle \,{\mathfrak {f}}_{0}^{},\,{\mathfrak {f}}_{\Psi }^{}({z})\big \rangle _{{{\mathcal {F}}}}^{}= {\big \langle \,{\mathfrak {f}}_{\textrm{out}}^{*},\, {\mathfrak {f}}_{\textrm{in}}^{}(z,\kappa )\,\big \rangle _{{{\mathcal {F}}}_\sigma }^{}}. \end{aligned}$$

The relation with conformal field theory is further developed in [CLT, Appendix G], where it is explained how the relation (5.23) can be derived using ideas from conformal field theory. It basically represents the free fermion conformal block ${\mathfrak {f}}_{\Psi }^{}({z})$ by the gluing construction from CFT associated to the decomposition of *C* into $C^{\textrm{in}}$ and $C^{\textrm{out}}$ described in Sect. 5.1.2. It is well-known (see e.g. [T17a]) that the gluing construction defines families of conformal blocks satisfying (4.18). It follows from (5.23) and (4.20) that

5.24 $$\begin{aligned} {\big \langle \,{\mathfrak {f}}_{\textrm{out}}^{*},\, {\mathfrak {f}}_{\textrm{in}}^{}(z,\kappa )\,\big \rangle _{{{\mathcal {F}}}_\sigma }^{}}=N(\mu ) {\mathcal {T}}(\mu ;z), \end{aligned}$$

with ${\mathcal {T}}(\mu ;z)$ being the isomonodromic tau-function.

### Factorisation expansions

It is furthermore explained in Appendix A how to represent the matrix element occurring in (5.23) in terms of the Fredholm determinant

5.25 $$\begin{aligned} {\mathcal {T}}\big (\sigma ,\kappa ;\,{\underline{\theta }};\,z\big ):=\frac{\big \langle \,{\mathfrak {f}}_{\textrm{out}}^{*},\, {\mathfrak {f}}_{\textrm{in}}^{}\,\big \rangle _{{{\mathcal {F}}}_\sigma }^{}}{\big \langle \,{\mathfrak {f}}_{\textrm{out}}^{*},\,{\mathfrak {f}}_0^{}\,\big \rangle _{{{\mathcal {F}}}_\sigma }^{} \big \langle \,{\mathfrak {f}}_0^{},\,{\mathfrak {f}}_{\textrm{in}}^{}\,\big \rangle _{{{\mathcal {F}}}_\sigma }^{}} = {\textrm{det}}(1+{\textsf{A}}^{\textrm{out}}{\textsf{A}}^{\textrm{in}}), \end{aligned}$$

with ${\textsf{A}}^{\textrm{in}}$ being the operator represented by the matrices ${A}^{\textrm{in}}_{kl}$ defined from $\Psi ^{\textrm{in}}_{q,\kappa }$ by first defining $A^{\textrm{in}}(x,y)$ from

5.26 $$\begin{aligned} \frac{(\Psi ^{\textrm{in}}_{q,\kappa }(x))^{-1}\Psi ^{\textrm{in}}_{q,\kappa }(y)}{x-y}= x^{-D}\left( \frac{{1}}{x-y}+A^{\textrm{in}}(x,y)\right) y^{D}, \end{aligned}$$

and then expanding $A^{\textrm{in}}(x,y)$ in a double series of the form (4.15). The operator ${\textsf{A}}^{\textrm{out}}$ is defined in an analogous way. According to (5.24) one may identify the function ${\mathcal {T}}(\sigma ,\kappa ;{\underline{\theta }};z)$ as the isomonodromic tau-function defined with a specific choice of normalisation condition. Representing ${\big \langle \,{\mathfrak {f}}_{\textrm{out}}^{*}\,,\, {\mathfrak {f}}_{\textrm{in}}^{}\,\big \rangle _{{{\mathcal {F}}}_\sigma }^{}}$ in terms of a Fredholm determinant makes it manifest, in particular, that ${\mathcal {Z}}_{\textrm{ff}}(\mu ,{\textbf{z}})$ is mathematically well-defined.

The determinants ${\textrm{det}}(1+{\textsf{A}}^{\textrm{out}}{\textsf{A}}^{\textrm{in}})$ can be expressed as sums over products of sub-determinants of the infinite matrices formed out of the matrices ${A}^{\textrm{in}}_{kl}$ and ${A}^{\textrm{out}}_{kl}$, respectively, see Appendix A.3 for more details. In this way it is not hard to see that in the case $C=C_{0,4}$ equation (5.25) yields series expansions of the following form:

5.27 $$\begin{aligned} {\mathcal {T}}\big (\sigma ,\kappa ;\,{\underline{\theta }};\,z\big ) = \sum _{n\in {{\mathbb {Z}}}} \,e^{in\kappa }\sum _{m=0}^\infty z^{m}\, {{\mathcal {R}}}_{n,m}(\sigma ,{\underline{\theta }}), \end{aligned}$$

where ${\underline{\theta }}=(\theta _1,\dots ,\theta _4)$. To understand this structure it may be useful to recall that the matrix elements ${A}^{\textrm{in}}_{kl}$ of ${\textsf{A}}^{\textrm{in}}$ are $2\times 2$-matrices in the case $N=2$ of our interest. It easily follows from the discussion in Sect. 5.1.2 together with (5.26) that the dependence of the $2\times 2$-matrices ${A}^{\textrm{in}}_{kl}$ on $\kappa$ is for all *k*, *l* given by the same factors $e^{\pm {\textrm{i}}\kappa }$ in the off-diagonal matrix elements of ${A}^{\textrm{in}}_{kl}$. It follows easily that the summation index *n* simply counts the difference of numbers of upper- and lower off-diagonal elements of matrices ${A}^{\textrm{in}}_{kl}$ in the sub-determinants appearing in the expansion of ${\textrm{det}}(1+{\textsf{A}}^{\textrm{out}}{\textsf{A}}^{\textrm{in}})$.

One should furthermore note that $\langle {\mathfrak {f}}_0^{},{\mathfrak {f}}_{\textrm{in}}^{}\big \rangle _{{{\mathcal {F}}}_\sigma }^{}$ has a dependence on *z* of the form

5.28 $$\begin{aligned} \big \langle \,{\mathfrak {f}}_0^{},\,{\mathfrak {f}}_{\textrm{in}}^{}\,\big \rangle _{{{\mathcal {F}}}_\sigma }^{}= N_{\textrm{in}}\,z^{\sigma ^2-\theta _1^2-\theta _2^2}, \end{aligned}$$

as follows from the relation between ${\mathfrak {f}}_{\textrm{in}}^{}$ and a conformal block on $C_{0,3}={\mathbb {P}}^1{\setminus }\{\infty ,z,0\}$ using the conformal Ward identities, $N_{\textrm{in}}$ being a constant.

The discussion in this section clarifies in particular how the normalisation factors $\nu _\epsilon ^{\textrm{out}}$ entering the definition of $\Psi ^{\textrm{out}}(x)$, $\Psi ^{\textrm{in}}(x)$ given in Sect. 5.1 via equation (5.6) determine unambiguously both (i) the precise definition of the variable $\kappa$ in (5.27), and (ii) how $\kappa$ is related to the coordinates for ${{\mathcal {M}}}_{\textrm{flat}}(C_{0,4})$ defined in Sect. 5.1. A canonical choice is of course $\nu _\epsilon ^{\textrm{out}}=1$ corresponding to the coordinates $(\sigma ,\kappa )$ defined in Sect. 5.1 using (5.4) together with formula (5.10) for $C_{}^{\pm }(\sigma )$. In this case one will get an expansion of the form (5.27) with coefficients ${{\mathcal {R}}}_{n,m}(\sigma ,{\underline{\theta }})$ which are rational functions of $(\sigma ,{\underline{\theta }})$. This follows easily from the fact that the matrix elements of ${A}^{\textrm{in}}_{kl}$ and ${A}^{\textrm{out}}_{kl}$ are assembled from the power series expansion coefficients of the hypergeometric function, which are rational functions of $(\sigma ,{\underline{\theta }})$.

#### Remark 2

The resulting picture is closely related to the one drawn in [GM, CGL]. Indeed, using the basic results from the theory of chiral free fermions summarised in Appendix A one may recognise the Fredholm determinants discussed in [CGL] as the free fermion matrix elements appearing here. A more direct proof that the Fredholm determinant on the right of (5.25) is the isomonodromic tau-function can be found in [CGL]. The normalisation prescription following from the definition (5.25) of the tau-functions is equivalent to the one used in [ILP].

## Representing Free Fermion Partition Functions as Generalised Theta-Series

The results of the last section imply that ${\mathcal {Z}}_{\textrm{ff}}(\sigma ,\kappa ;z)$ can be expanded as^11^

6.1 $$\begin{aligned} {\mathcal {Z}}_{\textrm{ff}}(\sigma ,\kappa ;z)=\sum _{n\in {{\mathbb {Z}}}} \,e^{in\kappa } {{\mathcal {F}}}_{n}(\sigma ,z). \end{aligned}$$

This expansion has a form consistent with the string duality conjectures discussed in [DHSV] only if the Fourier coefficients ${{\mathcal {F}}}_{n}(\sigma ,z)$ can represented by a function ${{\mathcal {F}}}(\sigma ,z)$ such that ${{\mathcal {F}}}_{n}(\sigma ,z)={{\mathcal {F}}}(\sigma +n,z)$. In that case one could expect that ${{\mathcal {F}}}(\sigma ,z)$ can be identified with the topological string partition function.

So far we had not fixed a normalisation for the states ${\mathfrak {f}}_{\textrm{out}}^{*}$ and ${\mathfrak {f}}_{\textrm{in}}^{}$, leaving the normalisation factors $N_{\textrm{out}}=\langle {\mathfrak {f}}_{\textrm{out}}^{*},{\mathfrak {f}}_0^{}\rangle _{{{\mathcal {F}}}_\sigma }^{}$ and $N_{\textrm{in}}=z^{\theta _1^2+\theta _2^2-\sigma ^2} \langle {\mathfrak {f}}_0^{},{\mathfrak {f}}_{\textrm{in}}^{}\rangle _{{{\mathcal {F}}}_\sigma }^{}$ entering the relation (5.25) between free fermion partition partition functions and Fredholm determinants arbitrary up to now. Considering generic choices for $N_{\textrm{out}}$ and $N_{\textrm{in}}$ we will observe that the free fermion partition functions ${\mathcal {Z}}_{\textrm{ff}}(\sigma ,\kappa ;z)$ do *not* admit series expansions of the desired form, in general.

The main goal in this section is to identify a small family of distinguished choices for $N_{\textrm{out}}\equiv N_{\textrm{out}}(\mu )$ and $N_{\textrm{in}}\equiv N_{\textrm{in}}(\mu )$ such that the free fermion partition functions ${\mathcal {Z}}_{\textrm{ff}}(\sigma ,\kappa ;z)$ admit series expansions of the required form. These choices will be in a direct correspondence with coordinates $(\sigma ,\kappa )$ of the type introduced in Sect. 5.1.

### Explicit form of the factorisation expansion

Explicit series expansions for the isomonodromic tau functions have first been conjectured in [GIL]. Proofs of this conjecture were given in [ILT, BS, GL16] by rather different methods. The proof closest to the formulation used in this paper is the one in [GL16]. It proceeds by explicit calculation of the determinant on the right side of (5.25) using an expansion as sum over sub-determinants. After stating the result we will discuss some of its features that will be important in the following.

The result of [GIL, ILT, BS, GL16] can be written as follows^12^

6.2 $$\begin{aligned} {\mathcal {T}}\big (\sigma ,\kappa ;\,{\underline{\theta }};\,z\big )= \sum _{n\in {{\mathbb {Z}}}} \,e^{in\kappa }\,F_n(\,\sigma ,\,{\underline{\theta }}\,) {{\mathcal {F}}}(\,\sigma +n,\,{\underline{\theta }};\,z\,), \end{aligned}$$

using the definitions

- The variables $\sigma$ and $\kappa$ are the coordinates for ${{\mathcal {M}}}_{\textrm{ch}}(C_{0,4})$ which are defined in Sect. 5.1 using (5.4) with the normalisation choice giving formula (5.10) for $C_{}^{\pm }(\sigma )$.
- The functions $F_n(\,\sigma ,\,{\underline{\theta }}\,)$ can be represented as
  $$\begin{aligned} F_n(\,\sigma \,,\,{\underline{\theta }}\,)&=\frac{\prod _{\epsilon ,\epsilon '=\pm } H_n(\sigma +\epsilon \theta _2+\epsilon '\theta _1) H_n(\sigma +\epsilon \theta _3+\epsilon '\theta _4)}{(H_{2n}(2\sigma ))^2}, \end{aligned}$$
  where $H_n(\sigma )$, $n\in {{\mathbb {Z}}}$, is the family of functions defined as
  6.3 $$\begin{aligned} H_n(\sigma )=\frac{G(1+\sigma +n)}{G(1+\sigma )(\Gamma (\sigma ))^n}, \end{aligned}$$
  with *G*(*p*) being the Barnes *G*-function satisfying $G(p+1)=\Gamma (p)G(p)$. Note that $F_n(\sigma ,{\underline{\theta }})$ are for all $n\in {{\mathbb {Z}}}$ *rational* functions of $\sigma$, as predicted by the discussion in Sect. 5.3.
- ${{\mathcal {F}}}(\,\sigma ,\,{\underline{\theta }};\,z\,)$ can be represented by a power series of the following form
  6.4 $$\begin{aligned} {{\mathcal {F}}}(\,\sigma ,\,{\underline{\theta }};\,z\,)=z^{\sigma ^2-\theta _1^2-\theta _2^2} (1-z)^{2\theta _2\theta _3}\sum _{\xi ,\zeta \in {\mathbb {Y}}}z^{|\xi |+|\zeta |}{{\mathcal {F}}}_{\xi ,\zeta }(\sigma ,{\underline{\theta }}), \end{aligned}$$
  where the summation is extended over pairs $(\xi ,\zeta )$ of partitions, and $|\xi |$ is the number of boxes in the Young diagram representing the partition $\xi$. The explicit formulae for the coefficients ${{\mathcal {F}}}_{\xi ,\zeta }(\sigma ,{\underline{\theta }})$ can be found in [GIL, GL16], where it is also observed that they are related to the instanton partition functions in the four-dimensional, ${{\mathcal {N}}}=2$-supersymmetric *SU*(2)-gauge theory with four flavors.

The normalisations in (6.2) are fixed such that ${{\mathcal {F}}}_{\emptyset ,\emptyset }(\sigma ,{\underline{\theta }})=1$.

### Rewriting as generalised theta series

The string dualities discussed in [DHSV] suggest that the relevant fermionic partition functions should admit an expansion taking the form (1.2) of a generalised theta series. Formula (6.2) is not of this form, the summand depends on the variable $\sigma$ not only in the combination $\sigma +n$. One may note, however, that there are two types of ambiguities involved in the definition of the partition functions in general, and in the form of its series expansion in particular:

- There is generically a normalisation freedom in the definition of partition functions. While the dependence w.r.t. the variable *z* is governed by Ward identities, mathematically expressed in the definition (3.22) of the isomonodromic tau-function, the normalisation ambiguities leave the freedom to multiply the tau-function by a function depending only on the monodromy data.
- The coefficients in the series expansions of tau-functions like (6.2) depend on the precise definition of the coordinate $\kappa$. A change of coordinates from $(\sigma ,\kappa )$ to $(\sigma ,\tau )$ with $\tau$ satisfying $e^{i\kappa }=e^{i\tau }D(\sigma ,{\underline{\theta }})$ would change the coefficients $F_n$ in (6.2) by a factor of $(D(\sigma ,{\underline{\theta }}))^n$.

By combining these observations we will find a renormalised version of the tau-functions which will indeed admit an expansion of generalised theta-series type.

To this aim let us note that the change of variables from the coordinates $(\sigma ,\kappa )$ defined through (5.4) with $C^\pm _{\textrm{r}}(\sigma )$ given in (5.10) to coordinates $(\sigma ,\tau )$ with $C^\pm _{}(\sigma )$ given in (5.12) is such that

6.5 $$\begin{aligned} e^{i\tau }= e^{i\kappa }\frac{(2\pi )^2(\Gamma (2\sigma ))^4}{ \prod _{s,s'=\pm 1}\Gamma (\sigma +s\theta _1+s' \theta _2\big )\Gamma (\sigma +s\theta _3+s' \theta _4)}. \end{aligned}$$

Rewriting (6.2) in terms of $\tau$ therefore yields the expansion

6.6 $$\begin{aligned} {\mathcal {T}}\big (\sigma ,\kappa ;\,{\underline{\theta }};\,z\big ):=\, \sum _{n\in {{\mathbb {Z}}}} \,e^{in\tau }\,G_n(\,\sigma ,\,{\underline{\theta }}\,) {{\mathcal {F}}}(\,\sigma +n,\,{\underline{\theta }};\,z\,), \end{aligned}$$

where the coefficients $G_n$ can be represented in the form

6.7 $$\begin{aligned} G_n(\,\sigma ,\,{\underline{\theta }}\,)=\frac{N(\sigma +n,\theta _4,\theta _3)N(\sigma +n,\theta _2,\theta _1)}{N(\sigma ,\theta _4,\theta _3)N(\sigma ,\theta _2,\theta _1)}, \end{aligned}$$

with

6.8 $$\begin{aligned} N(\theta _3,\theta _2,\theta _1)\,=\,\frac{\prod _{\epsilon ,\epsilon '=\pm }G(1+\theta _3+\epsilon \theta _2+\epsilon '\theta _1)}{G(1+2\theta _3)G(1+2\theta _2)G(1+2\theta _1)G(1)}. \end{aligned}$$

The structure of the right hand side of formula (6.6) now suggests to define

6.9 $$\begin{aligned} {\mathcal {Z}}\big (\sigma ,\tau ;\,{\underline{\theta }};\,z\big )= {N(\sigma ,\theta _4,\theta _3)N(\sigma ,\theta _2,\theta _1)}{\mathcal {T}}\big (\sigma ,\kappa ;\,{\underline{\theta }};\,z\big ), \end{aligned}$$

which can indeed be represented in the form of a theta-series.

6.10 $$\begin{aligned} {\mathcal {Z}}\big (\sigma ,\tau ;\,{\underline{\theta }};\,z\big )= \sum _{n\in {{\mathbb {Z}}}} \,e^{in\tau }\, {{\mathcal {G}}}(\,\sigma +n,\,{\underline{\theta }};\,z\,), \end{aligned}$$

with ${{\mathcal {G}}}(\sigma ,{\underline{\theta }};z)= {N(\sigma ,\theta _4,\theta _3)N(\sigma ,\theta _2,\theta _1)} {{\mathcal {F}}}(\sigma ,{\underline{\theta }};z).$ Equation (6.10) displays the form (1.2) expected for the topological string partition functions. It will turn out, however, that there still is some freedom in the choice of the function *N* representing the normalisation freedom. We are next going to describe this freedom, which turns out to be related to redefinitions of the coordinates $(\sigma ,\kappa )$. In order to get a precise match with the topological string partition functions we will have to identify the choices of coordinates $(\sigma ,\kappa )$ relevant for a given chamber in the extended Kähler moduli space. This will finally be achieved in Sect. 8.5.

### Alternative representations as theta series

In this subsection we will identify a small family of normalisation conditions defining tau-functions sharing the feature to admit an expansion as a theta-series of the form (1.2). We will observe that all normalisation conditions in this class are obtained from (6.10) by combining a redefinition of the normalising factors $N(\theta _3,\theta _2,\theta _1)$ with a modification of the definition of the coordinate $\tau$. Each such choice of normalisation thereby corresponds to a particular set of coordinates for the space of monodromy data.

To find alternatives to the expansion (6.10) let us consider the possibility to replace the functions $N(\theta _3,\theta _2,\theta _1)$ in (6.9) by functions $N'(\theta _3,\theta _2,\theta _1)$ such that, for example,

6.11 $$\begin{aligned} N(\theta _3,\theta _2,\theta _1)={\prod _{\epsilon =\pm }S(\theta _3+\epsilon (\theta _2-\theta _1))} N'(\theta _3,\theta _2,\theta _1), \end{aligned}$$

where *S*(*x*) is the special function $S(x)=(2\pi )^{-x}\frac{G(1+x)}{G(1-x)}.$ Noting that the function *S*(*x*) satisfies the functional relation

6.12 $$\begin{aligned} S(x\pm 1)=\mp \left( 2\sin \pi x \right) ^{\mp 1}S(x), \end{aligned}$$

we find the relation

6.13 $$\begin{aligned} \frac{N(\sigma +n,\theta _4,\theta _3)N(\sigma +n,\theta _2,\theta _1)}{N(\sigma ,\theta _4,\theta _3)N(\sigma ,\theta _2,\theta _1)}&=\frac{N'(\sigma +n,\theta _4,\theta _3)N'(\sigma +n,\theta _2,\theta _1)}{N'(\sigma ,\theta _4,\theta _3)N'(\sigma ,\theta _2,\theta _1)}\nonumber \\&\quad \times \prod _{\epsilon =\pm }\Big [2\sin \pi (\sigma +\epsilon (\theta _2-\theta _1))2\sin \pi (\sigma +\epsilon (\theta _4-\theta _3))\Big ]^{-n}. \end{aligned}$$

Introducing a new coordinate $\tau '=\tau (\sigma ,\tau )$ which is defined such that

6.14 $$\begin{aligned} e^{i\tau }&=e^{i\tau '}\,\prod _{\epsilon =\pm }\, {2\sin \pi (\sigma +\epsilon (\theta _1-\theta _2))}\, {2\sin \pi (\sigma +\epsilon (\theta _3-\theta _4))}, \end{aligned}$$

along with

6.15 $$\begin{aligned} {\mathcal {Z}}'\big (\sigma ,\tau '(\sigma ,\tau );\,{\underline{\theta }};\,z\big )= \frac{N(\sigma ,\theta _4,\theta _3)N(\sigma ,\theta _2,\theta _1)}{N'(\sigma ,\theta _4,\theta _3)N'(\sigma ,\theta _2,\theta _1)}\, {\mathcal {Z}}\big (\sigma ,\tau ;\,{\underline{\theta }};\,z\big ) \end{aligned}$$

we see that ${\mathcal {Z}}'(\sigma ,\tau ';{\underline{\theta }};z)$ also has a representation as a generalised theta series,

6.16 $$\begin{aligned} {\mathcal {Z}}'\big (\sigma ,\tau ';\,{\underline{\theta }};\,z\big )= \sum _{n\in {{\mathbb {Z}}}} \,e^{in\tau '}\, {{\mathcal {G}}}'(\,\sigma +n,\,{\underline{\theta }};\,z\,). \end{aligned}$$

It is clear the partition functions ${\mathcal {Z}}'(\sigma ,\tau ';{\underline{\theta }};z)$ and ${\mathcal {Z}}(\sigma ,\tau ;{\underline{\theta }};z)$ differ by a factor only depending on monodromy data, We conclude that a change of normalisation of the partition functions correlated with the change of coordinates $(\sigma ,\tau )\rightarrow (\sigma ,\tau ')$ may preserve the feature that the partition function can be represented as a generalised theta-series.

There are, of course, a few other options similar to (6.11) one might consider. It is natural to restrict attention to redefinitions of the function $N(\theta _3,\theta _2,\theta _1)$ in order to preserve a form of the expansion adapted to the pants decomposition it corresponds to. By redefinitions similar to (6.11) one can change the sign of the argument of each of the four *G*-functions appearing in (6.8), giving sixteen options in total.

The free fermion partition functions defined in the previous sections depend on the choice of normalisation factors $\nu _+^{\textrm{out}}$ and $\nu _-^{\textrm{in}}$ for the solutions to the Riemann–Hilbert problems on $C^{\textrm{out}}$ and $C^{\textrm{in}}$ introduced in Sect. 5.1, respectively. We have seen that the requirement that the free fermion partition functions admit an expansion of generalised theta-series type restricts the normalisation freedom considerably. Only very special choices of possibly monodromy-dependent normalisation factors have this property. However, the requirement to have a generalised theta series expansion does not fix the normalisation choice uniquely, there is a fairly small family of choices which all yield expansions of theta series type.

## Comparison with Topological Vertex Calculations

We will now compare our findings to an alternative computation of the topological string partition function which can be done with the help of the topological vertex [AKMV]. The topological string partition functions have been computed previously for the case of our interest in [IK03a, IK03b, EK, HIV, IK04]. A key issue for our goals is the dependence of the partition functions on the choice of a chamber.^13^ The chambers are related flop transitions. The changes of the partition functions associated to flop transitions were previously studied in [IK04, KM]. We will now summarise the relevant results.

The partition functions ${Z}^{\textrm{top}}_{{\mathfrak {i}},{\mathfrak {j}}}$ associated to the chambers ${\mathfrak {C}}_{{\mathfrak {i}},{\mathfrak {j}}}$ can be represented the form

7.1 $$\begin{aligned} {Z}^{\textrm{top}}_{{\mathfrak {i}},{\mathfrak {j}}}=z^{\sigma ^2-\theta _1^2-\theta _2^2} {Z}^{\textrm{out}}_{{\mathfrak {i}}}\, {Z}^{\textrm{in}}_{{\mathfrak {j}}}\, Z^{\textrm{inst}}_{}, \end{aligned}$$

The factor denoted $Z^{\textrm{inst}}$ in (7.1) is known in the literature as the five dimensional Nekrasov instanton partition function [N]. This part is independent of the choice of a chamber. Of main interest for us are the factors ${Z}^{\textrm{out}}_{{\mathfrak {i}}}$, ${Z}^{\textrm{in}}_{{\mathfrak {j}}}$. In the case $({\mathfrak {i}},{\mathfrak {j}})=({{\mathfrak {1}}},{{\mathfrak {1}}})$ corresponding to the toric diagram depicted in Fig. 1 we find, for example,

7.2 $$\begin{aligned} Z^{\textrm{out}}_{{{\mathfrak {1}}}} =\frac{{\mathcal {M}}(Q_F){\mathcal {M}}(Q_{3}Q_{4}Q_F)}{\prod _{i=3}^4{\mathcal {M}}\big (Q_{i}\big ){\mathcal {M}}\big (Q_{i}Q_F\big )}, \qquad Z^{\textrm{in}}_{{{\mathfrak {1}}}} =\frac{{\mathcal {M}}\big (Q_F\big ){\mathcal {M}}(Q_{1}Q_{2}Q_F)}{\prod _{i=1}^2{\mathcal {M}}\big (Q_{i}\big ){\mathcal {M}}\big (Q_{i}Q_F\big )}. \end{aligned}$$

${\mathcal {M}}(Q)$ in (7.2) is defined as ${\mathcal {M}}(Q) \equiv (Q q;q, q)_{\infty }^{-1}$, with $q=e^{-\lambda R}$ and $(Q;t, q)_{\infty }$ being

7.3 $$\begin{aligned} (Q;t, q)_{\infty }= \prod _{i,j=0}^{\infty }(1-Q t^{i} q^j) \quad \text { for }\; |t|<1,\; | q|<1. \end{aligned}$$

**Fig. 3:**
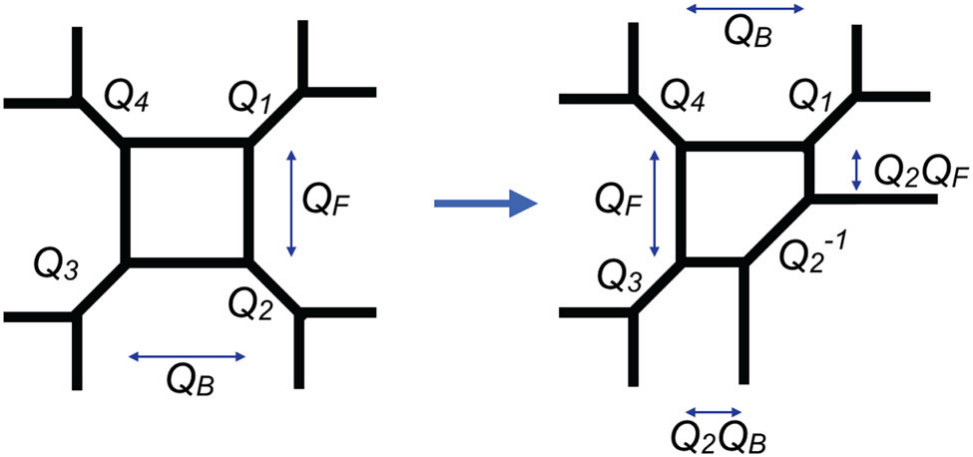
Two toric diagrams related via flopping both engineering *SU*(2) superconformal QCD with $N_f=4$ fundamental hypermultiplets

An example for a pair of toric diagrams related by a flop is depicted in Fig. 3. The effect of flops on the partition functions can be described by the relations

7.4 $$\begin{aligned} \begin{aligned} {Z}^{\textrm{in}}_{{{\mathfrak {2}}}}&= {{\mathcal {R}}}(Q_2)\,Z^{\textrm{in}}_{{{\mathfrak {1}}}}, \\ {Z}^{\textrm{in}}_{{{\mathfrak {3}}}}&= {{\mathcal {R}}}(Q_1)\,Z^{\textrm{in}}_{{{\mathfrak {2}}}}, \end{aligned} \qquad \begin{aligned} {Z}^{\textrm{out}}_{{{\mathfrak {2}}}}&= {{\mathcal {R}}}(Q_3)\,Z^{\textrm{in}}_{{{\mathfrak {1}}}}, \\ {Z}^{\textrm{out}}_{{{\mathfrak {3}}}}&= {{\mathcal {R}}}(Q_4)\,Z^{\textrm{in}}_{{{\mathfrak {2}}}}, \end{aligned} \qquad {{\mathcal {R}}}(Q)=\frac{{\mathcal {M}}(Q)}{{\mathcal {M}}(Q^{-1})} . \end{aligned}$$

We have thereby defined $Z^{\textrm{top}}$ as a piecewise analytic function on the union of the chambers ${\mathfrak {C}}_{ {\mathfrak {i}}{\mathfrak {j}}}$.

Taking the limit $R\rightarrow 0$ of the functions ${Z}^{\textrm{top}}_{{\mathfrak {i}},{\mathfrak {j}}}$ with fixed $m_1,\dots ,m_4,a,\lambda$ is delicate as the functions ${{\mathcal {M}}}(Q)$ diverge in this limit. In order analyse the limit we may use the relation

7.5 $$\begin{aligned} \frac{{{\mathcal {M}}}(q^u)}{{{\mathcal {M}}}(1)}=\frac{(q;q)_\infty ^{u}(1-q)^{-\frac{1}{2}(u-1)u}}{G_q(1+u)}, \end{aligned}$$

with $G_q(u)$ being the q-Barnes function which has a limit $\lim _{q\rightarrow 1}G_q(u)=G(u)$ [UN]. Note that $(q;q)_\infty$ behaves as $(q;q)_\infty \sim e^{-\frac{\pi {\textrm{i}}}{12\tau }} (-i\tau )^{-\frac{1}{2}}$ for $q=e^{2\pi {\textrm{i}}\tau }$, $\tau \rightarrow 0$.

However, there exist natural renormalisation prescriptions allowing us to define meaningful quantities ${{{\mathcal {Z}}}}^{\textrm{top}}_{{\mathfrak {i}},{\mathfrak {j}}}$ which can be associated to ${Z}^{\textrm{top}}_{{\mathfrak {i}},{\mathfrak {j}}}$ in the limit $R\rightarrow 0$. Applying (7.5) for the analysis of the limit of ${Z}^{\textrm{top}}_{{\mathfrak {i}},{\mathfrak {j}}}$ is simplest for ${\mathfrak {i}}={\mathfrak {j}}=2$. Using (7.5) one may show that

7.6 $$\begin{aligned} {{\mathcal {Z}}}^{\textrm{top}}_{{{\mathfrak {2}}},{{\mathfrak {2}}}}=\lim _{R\rightarrow 0} {\tilde{Z}}^{\textrm{top}}_{{{\mathfrak {2}}},{{\mathfrak {2}}}},\qquad {\tilde{Z}}^{\textrm{top}}_{{{\mathfrak {2}}},{{\mathfrak {2}}}}:={{\mathcal {M}}}(e^{-2Ra_2}){{\mathcal {M}}}(e^{-2Ra_3})({{\mathcal {M}}}(1))^2\, {Z}^{\textrm{top}}_{{{\mathfrak {2}}},{{\mathfrak {2}}}}, \end{aligned}$$

exists. It follows from the existence of a limit for ${\tilde{Z}}^{\textrm{top}}_{{{\mathfrak {2}}},{{\mathfrak {2}}}}$ that the singular behavior of ${Z}^{\textrm{top}}_{{{\mathfrak {2}}},{{\mathfrak {2}}}}$ does not depend on the variables $\sigma =-\frac{1}{\lambda R}\log Q_F$ and $z=Q_B$. While there do exist alternatives for the definition of finite quantities from ${Z}^{\textrm{top}}_{{{\mathfrak {2}}},{{\mathfrak {2}}}}$ in the limit $R\rightarrow 0$, it is unnecessary and unnatural to introduce extra factors altering the dependence on $\sigma$ and *z* in the definition of this limit. A more detailed discussion of these issues can be found in [CPT19].

This motivates us to define the four-dimensional limit of $Z^{\textrm{top}}_{{{\mathfrak {2}}},{{\mathfrak {2}}}}$ as follows:

7.7 $$\begin{aligned} {{\mathcal {Z}}}^{\textrm{top}}_{{{\mathfrak {2}}},{{\mathfrak {2}}}}(\,a,\,{\underline{m}};\,z\,)= {{\mathcal {Z}}}^{\textrm{out}}_{{{\mathfrak {2}}}}\,{{\mathcal {Z}}}^{\textrm{in}}_{{{\mathfrak {2}}}}\, {{\mathcal {F}}}(\,\sigma ,\,{\underline{\theta }};\,z\,), \end{aligned}$$

where ${{\mathcal {F}}}(\,\sigma ,\,{\underline{\theta }};\,z\,)$ is defined in (6.4), the Kähler parameters of *X* are related to $\sigma$ and $\theta _1,\dots ,\theta _4$ as $\sigma =a/\lambda$, $\theta _i=m_i/\lambda ,$ for $i=1,\dots ,4$, and ${{\mathcal {Z}}}^{\textrm{out}}_{{{\mathfrak {2}}}}=M(\sigma ,\theta _4,\theta _3)$, ${{\mathcal {Z}}}^{\textrm{in}}_{{{\mathfrak {2}}}}=M(\sigma ,\theta _2,\theta _1)$ with

$$\begin{aligned} M(\theta _3,\theta _2,\theta _1)=\frac{ \prod _{s=\pm }G(1+s\theta _3+\theta _2+\theta _1)G(1+\theta _3+s(\theta _2-\theta _1))}{G(1+2\theta _3)G(1+2\theta _2)G(1+2\theta _1)G(1)}. \end{aligned}$$

It seems furthermore natural to extend the the definition of ${{{\mathcal {Z}}}}^{\textrm{top}}_{{\mathfrak {i}},{\mathfrak {j}}}$ to all ${\mathfrak {i}},{\mathfrak {j}}={{\mathfrak {1}}},{{\mathfrak {2}}},{{\mathfrak {3}}}$ by demanding that we have a natural analog of the relations (7.4) describing the effects of flops,

7.8 $$\begin{aligned} \begin{aligned} {{{\mathcal {Z}}}}^{\textrm{in}}_{{{\mathfrak {2}}}}&= R_\rho (Q_2)\,{{\mathcal {Z}}}^{\textrm{in}}_{{{\mathfrak {1}}}}, \\ {{{\mathcal {Z}}}}^{\textrm{in}}_{{{\mathfrak {3}}}}&= R_\rho (Q_1)\,{{\mathcal {Z}}}^{\textrm{in}}_{{{\mathfrak {2}}}}, \end{aligned} \qquad \begin{aligned} {{{\mathcal {Z}}}}^{\textrm{out}}_{{{\mathfrak {2}}}}&= R_\rho (Q_3)\,Z^{\textrm{in}}_{{{\mathfrak {1}}}}, \\ {{{\mathcal {Z}}}}^{\textrm{out}}_{{{\mathfrak {3}}}}&= R_\rho (Q_4)\,Z^{\textrm{in}}_{{{\mathfrak {2}}}}, \end{aligned} \end{aligned}$$

with $R_\rho (x)=\rho ^{x}\frac{G(1-x)}{G(1+x)}$ being a renormalised version of the limit $q\rightarrow 1$ of ${{\mathcal {R}}}(q^x)$. The factor $\rho ^{x}$ reflects the usual ambiguity in the renormalisation of $(1-q)^x= (1-e^{-\lambda R})^x$ for $R\rightarrow 0$ resulting from the possibility to redefine $R\rightarrow \rho R$.

The results display a simple pattern. The normalisation factors ${{\mathcal {Z}}}^{\textrm{in}}$ and ${{\mathcal {Z}}}^{\textrm{out}}$ can be one of the functions $N_i=N_i(\vartheta _3,\vartheta _2,\vartheta _1)$, $i=1,2,3$, defined as

7.9 $$\begin{aligned} N_i(\vartheta _3,\vartheta _2,\vartheta _1)=\frac{ \prod _{s,s'=\pm }G(1+\vartheta _i+s\vartheta _{i+1}+s'\vartheta _{i+2})}{G(1+2\vartheta _3)G(1+2\vartheta _2)G(1+2\vartheta _1)G(1)}, \qquad \vartheta _{i+3}\equiv \vartheta _i, \end{aligned}$$

or $N_s(\vartheta _3,\vartheta _2,\vartheta _1)=M(\vartheta _3,\vartheta _2,\vartheta _1)$. The functions $N_i$, $i=1,2,3,s$, are assigned to the region in the parameter space under consideration in such a way that the arguments in the *G*-functions appearing in the expressions for the functions $N_i$ are *always positive*.

Remembering the definition of the chambers ${\mathfrak {C}}_{{\mathfrak {i}},{\mathfrak {j}}}$, and taking into account the relations $\sigma =a/\lambda$ and $\theta _i=m_i/\lambda$ one finds for each chambers ${\mathfrak {C}}_{{\mathfrak {i}},{\mathfrak {j}}}$ a unique region defined by the conditions that all of the arguments of the functions $N_i$, $i=1,2,3,s$, appearing in the expression for ${{{\mathcal {Z}}}}^{\textrm{top}}_{{\mathfrak {i}},{\mathfrak {j}}}$ are positive for all values of $\lambda$. This becomes a one-to-one correspondence if one extends the topological vertex results to the cases where $a_2>a_1$ and $a_3>a_4$ not considered yet by the necessary modifications.

Comparing with the discussion in Sect. 6 we may finally observe that the partition functions characterised by these normalisation factors all represent Fourier coefficients in the generalised theta series expansions of suitably normalised free fermion partition functions. Keeping in mind that there was a direct correspondence between the normalisation factors defining such partition functions on the one hand, and choices of Darboux coordinates on the other hand, makes it natural to ask if there is a simple relation between the chambers in the parameter space considered above and the relevant choices of Darboux coordinates.

## Abelianisation

In Sect. 6 we had observed a direct relation between choices of coordinates for ${{\mathcal {M}}}_{\textrm{flat}}(C_{0,4})$ and normalisations of the tau-functions. It was next found that the theta series coefficients of tau-functions for certain choices of coordinates are equal to the topological string partition functions for the local CY manifolds of class $\Sigma$. These functions change from chamber to chamber in the complex structure moduli space. We will now see that there is a natural way to describe the dependence on the choice of a chamber. It turns out that the relevant coordinates for ${{\mathcal {M}}}_{\textrm{flat}}(C)$ can be defined using a construction introduced in [GMN12] and further developed in [HN]. Following [HN] we will refer this construction as abelianisation. We will begin this section with a brief review of this construction following [HN, HK].

### Spectral Networks

The curves $\Sigma$ defined in Subsection 2.1 as covers of Riemann surfaces *C* were specified by quadratic differentials $q=q(x)d^2 x$ in equations (2.2)-(2.4). The *spectral network* ${\mathcal {W}}_\theta (q)$ for *q* is a graph on *C* having oriented edges called *walls* which are defined by $\text {Im}\left( e^{i\theta }\int _a^x\sqrt{q(x')}dx'\right) =0$ and which meet at vertices, the zeros *a* of *q*. Three walls emanate from the branch points where two sheets meet. It is useful to choose a set of branch cuts on *C* and labels for the sheets of $\Sigma$, such that each wall of the network is labeled by an ordered pair of integers *ij* corresponding to the sheets. Given a positively oriented tangent vector *v* to the wall and $\theta \in {\mathbb {R}}/2\pi {\mathbb {Z}}$, the wall carries the label *ji* if $e^{-i\theta } (y_i-y_j)(v)\in {\mathbb {R}}_+$ and *ij* if $e^{-i\theta } (y_i-y_j)(v)\in {\mathbb {R}}_-$. For special values of *q* and $\theta$, two walls *ij* and *ji* can overlap and create a double wall. When this occurs there exist two possible *resolutions*, which are the infinitesimal ways of displacing the walls with respect to each other. When *q* obeys the Strebel condition $\oint _{\gamma _i}\sqrt{q(x)}dx\in {\mathbb {R}}_+$ on curves $\{\gamma _i\}$ defining a pants decomposition of *C*, the corresponding spectral network is a *Fenchel-Nielsen (FN) network* and composed of double walls only. A FN-network defines a pants decomposition of *C*, since its restriction to every three-punctured sphere in this decomposition is a FN-network.

**Fig. 4:**
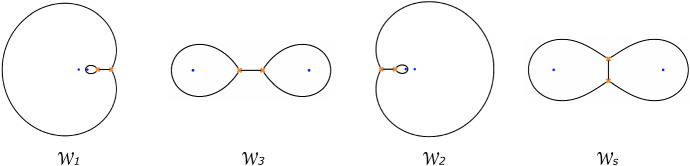
FN-networks on $C_{0,3}$, with punctures depicted at positions $z=0,1$. These isotopies occur in different regions of the parameter space and correspond to the possible choices of chamber ${\mathfrak {C}}_{\alpha }$ in Sect. 2.3. Network ${\mathcal {W}}_1$ corresponds to chamber ${\mathfrak {C}}^{\textrm{in}}_{{\mathfrak {1}}}$, while ${\mathcal {W}}_3$ corresponds to ${\mathfrak {C}}^{\textrm{in}}_{{\mathfrak {3}}}$ and ${\mathcal {W}}_s$ to ${\mathfrak {C}}^{\textrm{in}}_{{\mathfrak {2}}}$. The triplets of parameters $\{a_1,a_2,a\}$ in equation (2.4) take here the values: ${\mathcal {W}}_1$) $\{$0.51, 0.32, 0.18$\}$, ${\mathcal {W}}_3$) $\{$0.49, 0,48, 1$\}$ ${\mathcal {W}}_2$) $\{$0.32, 0.51, 0.18$\}$ and ${\mathcal {W}}_s$) $\{$0.51, 0.5, 1$\}$

When the base curve is $C_{0,3}={\mathbb {P}}^1\backslash \{0,1,\infty \}$, the quadratic differential defining the branched cover $\Sigma \rightarrow C_{0,3}$ was given in equation (2.4). There exist two inequivalent types of FN-networks: ${\mathcal {W}}_i$, $i=1,2,3$, are called type II molecules in Fig. 4 and ${\mathcal {W}}_s$ is a type I molecule.^14^ For each of these topologies, there is a choice for the resolution: *British*, where the outer walls of the network are oriented clockwise or *American*, for counter-clockwise orientation. We will here focus on the case where all the parameters *a* are real. Branch points will either be real, or come in complex conjugate pairs. The transitions between different types of molecules occur when two branch points coalesce, corresponding to the flop transitions discussed in Sect. 2.3. The branch points $t_{\pm }$ are easily read off from equation (2.11) and show that flop transitions occur when $a^2=(a_1+ a_2)^2$ and $a^2=(a_1- a_2)^2$. Therefore a molecule changes its isotopy class when *a* crosses any of the planes $a=\pm a_1\pm a_2$. Comparing with Sect. 2 one may note that such changes directly correspond to changes between the chambers in the complex structure moduli space defined there.

On general Riemann surfaces *C*, FN-networks can be defined with respect to pants decompositions found by gluing together molecules in the same resolution on the individual pants.

### ${\mathcal {W}}$-framed flat connections on *C*

The construction called abelianisation uses the spectral networks defined by a quadratic differential *q* to construct a natural one-to-one correspondence between flat *SL*(2)-connections on *C* and an (almost-)flat *GL*(1)-connections on the two-fold cover $\pi :\Sigma \rightarrow C$ defined by the differential *q*. Describing $\Sigma$ as a branched cover of *C* will then allow us to define ${\textrm{GL}}(1)$-connections $\nabla ^{\textrm{ab}}$ on $\Sigma$ from which one can recover all flat ${\textrm{SL}}(2)$-connections $\nabla$ on *C* by the construction sketched below. We are here interested in flat *SL*(2)-connections $\nabla$ in a complex vector bundle *E* over a Riemann surface *C* with fixed conjugacy class represented by $D_k = \text {diag}(e^{2\pi i \theta _k}, e^{-2\pi i \theta _k})$ at the $k^\text {th}$ puncture.

**Fig. 5:**
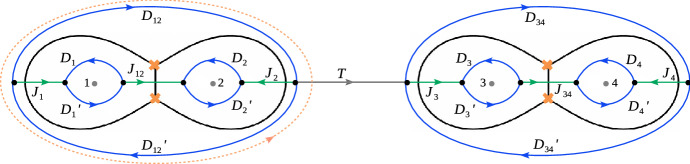
Fenchel-Nielsen network on the four-punctured sphere

We shall focus on the the cases where the spectral network has type FN. A FN-network ${\mathcal {W}}$ decomposes *C* into annular regions $A_i$ within which $\nabla$ can be diagonalised. Let ${\mathcal {G}}_C$ be the set of paths $\wp$ up to homotopy between base points marked by black dots in the example depicted in Fig. 5 on each side of the walls $w\in {\mathcal {W}}$. A path $\wp \in {\mathcal {G}}_C$ is “short" if it does not cross any walls.

In order to construct $\nabla$ from the connection $\nabla ^{\textrm{ab}}$ on $\Sigma$ one may start by considering the connection $\pi _*\nabla ^{\textrm{ab}}$ on the surface $C'$ obtained from *C* by removing the branch points of $\Sigma$. Choosing a basis $(s_1,s_2)$ of *E* at any point on $C\backslash {\mathcal {W}}$, the parallel transport of $(s_1,s_2)$ with respect to $\pi _*\nabla ^{\textrm{ab}}$, with $\pi :\Sigma \rightarrow C$ being the covering projection, along short paths $\wp \subset C\backslash {\mathcal {W}}$ is represented by: i) a matrix $D_\wp =\text {diag}(d_\wp ,d_\wp ^{-1})$ for $\wp$ not crossing a branch cut within a pair of pants, ii) a matrix $${\tilde{D}}_{\wp }=D_{\wp } \big ({\begin{matrix} 0 &{} 1 \\ -1 &{} 0 \end{matrix}}\big )$$ for $\wp$ intersecting a branch cut from a simple branch point, and iii) a matrix $T_\wp =\text {diag}(e^{\pi i\eta },e^{-\pi i\eta })$ for $\wp$ traversing an annulus between pairs of pants. The data $d_\wp$ and $\eta$ characterise a flat abelian connection $\nabla ^{\textrm{ab}}$ on $\Sigma '{{\setminus }} \pi ^{-1}({\mathcal {W}})$, with $\Sigma '$ being the complement of the branch points of $\Sigma$. It has been observed in [HN] that $\nabla ^{\textrm{ab}}$ automatically extends over $\Sigma '$.

The connection $\pi _*\nabla ^{\textrm{ab}}$ is almost-flat in the sense that the holonomy around any branch point is $-1$. The freedom in the choice of the matrices $D_\wp$ is constrained by the conditions fixing the holonomies around the boundary components. The remaining freedom in the choice of the parameters $d_\wp$ is related to the abelian gauge freedom at the base points. We will describe this in two relevant examples below.

The abelian connection $\nabla ^{\textrm{ab}}$ can be turned into a non-abelian connection $\nabla$ by replacing all products of matrices representing holonomies of $\pi _{*}\nabla ^{\textrm{ab}}$ by products obtained by splicing in certain triangular matrices for each segment of the path crossing a wall. Across a single wall, the non-abelian parallel transport of $(s_1,s_2)$ is represented by a triangular jump matrix *J*, whose precise form depends on the decoration assigned to the wall [HK]. The off-diagonal entries are determined uniquely in terms of the matrices $D_\wp$ by the consistency conditions stating that for every path which is contractible to a turning point (marked in orange in Fig. 5), the parallel transport is represented by the identity matrix [HN]. More details are given below for the cases of our interest.

Note that the map from the path groupoid ${\mathcal {G}}_C$ to the corresponding ${\textrm{SL}}(2)$ matrices is an anti-homomorphism. For the composition $\wp =\wp _1\wp _2$ of a path $\wp _1$ from point $i_1$ to $i_2$ with a path $\wp _2$ from $i_2$ to $i_3$ one multiplies the holonomy matrices $H_{\wp _1}$ and $H_{\wp _2}$ as $H_\wp =H_{\wp _2}H_{\wp _1}$.

### Four-punctured sphere: an example

We will now review how abelianisation works in the case $C=C_{0,4}$, following the previous discussions in [HN, HK]. Our goal will be to exhibit the residual ambiguities in the definition of FN-type coordinates, and to discuss natural ways to fix them.

**Fig. 6:**
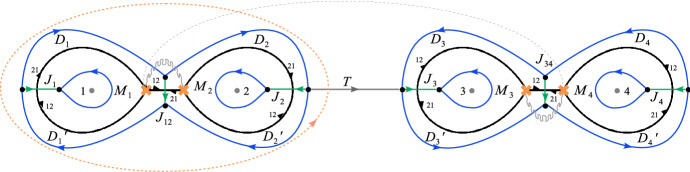
FN-network on $C_{0,4}$, formed by type-II molecules in the British resolution

As a first example we shall consider the network depicted in Fig. 6.

The ${\textrm{GL}}(1)$-connection defined on the cover $\Sigma$ of *C* is fully characterised by the matrices $D_\varpi$ associated to the blue paths $\varpi$ in Fig. 6.

Fixing the monodromy around the punctures implies relations of the form

8.1 $$\begin{aligned} M_i = \bigg ( \begin{matrix} {m}_i &{} 0 \\ 0 &{} {m}_i^{-1} \end{matrix} \bigg ) ~, \quad i=1,2, \quad D_2 D_1 D_1' D_2' = \bigg ( \begin{matrix} {m}_\alpha ^{-1} &{} 0 \\ 0 &{} {m}_\alpha \end{matrix} \bigg )~, \end{aligned}$$

with ${m}_i=e^{2\pi {\textrm{i}}\theta _i}$ for $i=1,2,3,4$ and ${m}_\alpha =e^{2\pi {\textrm{i}}\sigma }$. Parameterising the matrices $D_i$, $D_i'$ by complex numbers $d_i$, $d_i'$ for $i=1,2,3,4$ leads to the relation $d_1'd_2d_2'/d_1=-{m}_\alpha ^{-1}$, and a similar relation for $d_i$, $d_i'$, $i=3,4$. There is an obvious ambiguity in the choice of the parameters $d_i$, $i=1,2,3,4$, related to the freedom in the choice of the trivialisations at the base points.

This ambiguity affects the definition of FN-type coordinates. Assuming an arbitrary choice of the parameters $d_i$, $d_i'$, $i=1,2,3,4$ it is straightforward to find the corresponding FN-type coordinates as follows. In order to reconstruct the non-abelian ${\textrm{SL}}(2)$-connection $\nabla$ on *C* from the given ${\textrm{GL}}(1)$-connection $\nabla ^{\textrm{ab}}$ one mainly needs to find the jump matrices associated to the green paths crossing the walls. The jump matrices $J_1$, $J_2$, $J_{12}$ associated to the left part of the network in Fig. 6 will be parameterised as

8.2 $$\begin{aligned} J_i=\bigg ( \begin{matrix} 1 &{} 0 \\ {\tilde{c}}_i &{} 1 \end{matrix} \bigg ) \bigg ( \begin{matrix} 1 &{} c_i \\ 0 &{} 1 \end{matrix} \bigg ) ~, \quad i=1,2,\quad J_{12}=\bigg ( \begin{matrix} 1 &{} c_{12} \\ 0 &{} 1 \end{matrix} \bigg ) \bigg ( \begin{matrix} 1 &{} 0 \\ {\tilde{c}}_{12} &{} 1 \end{matrix} \bigg )~. \end{aligned}$$

These matrices are required to satisfy the following constraints

8.3 $$\begin{aligned} D_1' J_{12} D_1 J_1^{-1} M_1 J_1 = \textbf{1} ~, \quad D_2 J_{12}^{-1} D_2' J_2^{-1} M_2 J_2 = \textbf{1} ~. \end{aligned}$$

The constraints determine the parameters of the jump matrices uniquely in terms of the elements of the *D*-matrices. The resulting expressions are

8.4 $$\begin{aligned} c_1&= \frac{d_1d_1' ({m}_1 {m}_2 -{m}_\alpha )({m}_1-{m}_2 {m}_\alpha )}{{m}_1 {m}_2 \left( 1-{m}_\alpha ^2\right) } ~, \qquad {\tilde{c}}_1 = \frac{{m}_1}{d_1d_1' \left( 1- {m}_1^2\right) } \nonumber \\ c_2&= \frac{d_2 ({m}_1 {m}_2-{m}_\alpha ) ({m}_1 {m}_\alpha -{m}_2)}{d_2' {m}_1 {m}_2 \left( {m}_\alpha ^2-1\right) } ~, \qquad {\tilde{c}}_2 = \frac{d_2' {m}_2}{d_2\left( 1- {m}_2^2\right) } \nonumber \\ c_{12}&= \frac{d_2 d_2' {m}_\alpha \left( {m}_1 {m}_\alpha \left( 1+{m}_2^2\right) - {m}_2 \left( 1+{m}_1^2\right) \right) }{{m}_1 {m}_2 \left( {m}_\alpha ^2-1\right) } ~, \nonumber \\ {\tilde{c}}_{12}&= \frac{{m}_1(1+{m}_2^2) - {m}_2 {m}_\alpha (1+{m}_1^2) }{d_2 d_2' {m}_1 {m}_2 \left( {m}_\alpha ^2-1 \right) } ~. \end{aligned}$$

Determining the jump matrices $J_3$, $J_4$, $J_{34}$ associated to the right part of the network in Fig. 6 in a similar way, and representing the abelian parallel transport along the grey path connecting the two pairs of pants by the matrix $T=\text {diag}(e^{\pi i\eta },e^{-\pi i\eta })$, yields a parameterisation of the non-abelian ${\textrm{SL}}(2)$-connections $\nabla$ on *C* through the data determining $\nabla ^{\textrm{ab}}$. The clockwise monodromy around the punctures 2 and 3 is

8.5 $$\begin{aligned} U = T^{-1} M_3^\text {out} T M_2^\text {in} = T^{-1} J_3^{-1} M_3 J_3 T J_2^{-1} M_2 J_2 ~, \end{aligned}$$

The matrix *U* has trace

8.6 $$\begin{aligned} L_u = P_+^{-1} + N_0 + P_+ N ~, \qquad P_+ = \frac{d_2}{d_2'd_3d_3'} e^{2\pi i \eta } ~, \end{aligned}$$

where the parameters $d_i$, $d_i'$, $i=1,2,3,4$, obey $d_1'd_2d_2'/d_1=-e^{-2\pi i \sigma }=d_3d_4d_4'/d_3'$. The remaining coefficients in equation (8.6) are

8.7 $$\begin{aligned} (2 \sin (2\pi \sigma ))^2 N_0&= - 2\left[ \cos 2\pi \theta _1 \cos 2\pi \theta _4 + \cos 2\pi \theta _2 \cos 2\pi \theta _3 \right] \nonumber \\&\quad + 2\cos 2\pi \sigma \left[ \cos 2\pi \theta _1 \cos 2\pi \theta _3 + \cos 2\pi \theta _2 \cos 2\pi \theta _4 \right] , \end{aligned}$$

and

8.8 $$\begin{aligned} (2 \sin (2\pi \sigma ))^4 N = \prod _{s,s'=\pm 1} 2\sin \pi (\sigma +s\theta _1+s'\theta _2) 2\sin \pi (\sigma +s\theta _3+s'\theta _4) ~. \end{aligned}$$

Equation (8.6) exhibits clearly how the gauge freedom in the description of the connection $\nabla ^{\textrm{ab}}$ affects the definition of the coordinate $\eta$.

In order to discuss natural ways for fixing this freedom let us note that the freedom is related to the fact that the annular region between the two pairs of pants is not simply connected. Introducing extra cuts can produce simply-connected regions within which the ${\textrm{GL}}(1)$-connection can be trivialised. The holonomy of the corresponding ${\textrm{SL}}(2)$-connection around the annular region *A* and the parallel transport *T* from one boundary to the other are the data characterising the connection $\nabla$ in *A*. One may, however, choose to factorise this holonomy around *A* into as a product over contributions associated to paths crossing the cuts decomposing *A* into simply-connected pieces. The resulting freedom can be represented by the choice of $d_i$, $d_i'$, $i=1,2,3,4$. However, this point of view suggests that the remaining freedom in the definition of the FN-type coordinate $\eta$ represented by the factor $\frac{d_2}{d_2'd_3d_3'}$ in (8.6) can be written as the exponential of a linear function in the variable $\sigma$.

A few options are clearly distinguished by their simplicity. One option is to use a single extra cut to decompose *A*, an example is indicated in an example Fig. 6 by the dashed grey line. The discussion in the previous paragraph would suggest the choice $d_1=d_4=-e^{2\pi i \sigma }$, all other $d_i$, $d_i'$, $i=1,2,3,4$ being equal to unity. In this case one would simply find $P_+ = e^{2\pi i \eta }$. Other simple options amount to having $d_2=d_2'$ and $d_3=1/d_3'$, also leading to $P_+ = e^{2\pi i \eta }$. One should notice that the FN-type coordinates $(\sigma ,\eta )$ defined by these choices are related to the coordinates $(\sigma ,\kappa )$ appearing in the generalised theta series (6.2) by $\kappa =2\pi \eta$.

### Four-punctured sphere: the other cases

**Fig. 7:**
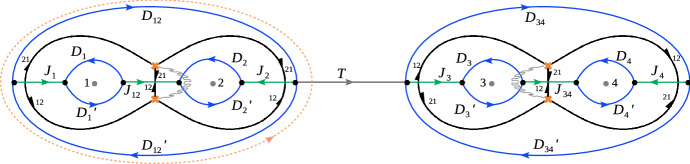
FN-network on $C_{0,4}$, formed by type-I molecules in the British resolution

Another interesting case is associated to the network depicted in Fig. 7.

The *D*-matrices which describe parallel transport along the blue paths $\wp$ in Fig. 7 obey relations $D_1D_1'=M_1$, $D_2'D_2=M_2$, $D_{12}D_{12}'=S^{-1}$ and similarly for the molecule on the right side. In this case there are a few natural options for fixing the residual freedom left by these constraints. A first option is to choose one of the two matrices $D_i$, $D_i'$ to be equal to the identity for $i=1,2,12,3,4$. However, in there would still be a discrete family of choices left. Most natural appears to be the choice $D_i=D_i'=\sqrt{M}_i$, for $i=1,2,12,3,4$. This is also the case which is most symmetric.

With this choice one may proceed with the construction of the connections $\nabla ^{\textrm{ab}}$ and $\nabla$ as explained in the previous section. The monodromy around the orange curve is $S=\text {diag}(e^{2\pi i \sigma },e^{-2\pi i \sigma })$ and the clockwise monodromy around the punctures 2 and 3 is

8.9 $$\begin{aligned} U = T^{-1} M_3^{\mathrm{\scriptscriptstyle out}} \,T\, M_2^{\mathrm{\scriptscriptstyle in}} = T^{-1} J_3^{-1} M_3 J_3 T J_2^{-1} M_2 J_2 ~. \end{aligned}$$

The matrix *U* has trace of the form $L_u = {\tilde{P}}_+^{-1} + N_0 + {\tilde{P}}_+ N$ with

$$\begin{aligned}&{\tilde{P}}_+ = - 4 \, \sin \pi (\sigma -\theta _1-\theta _2) \, \sin \pi (\sigma -\theta _3-\theta _4) \, e^{2\pi i \eta } ~, \end{aligned}$$

the coefficients $N_0$ and *N* being the same as in (8.7) and (8.8). Switching the resolution of the network in Fig. 7 is equivalent to replacing $P_+$ by $P_+^{-1}$.

It is then straightforward to treat the remaining cases in a similar way. In Subsection 8.1 we had observed a correspondence between flop transitions and changes of topological type of the FN-networks. It turns out that a set of simple rules describes the effect of flop transitions on the coordinates defined by abelianisation. In this way one finds a simple set of rules for the changes of FN-coordinates induced by flop transitions, the result of which is summarised in Table 1.

**Table 1:** The table collects the changes of FN-type coordinates associated to changes of the part of the network in the pair of pants containing punctures 1 and 2. The factors given in the lower row table are the ratios between $e^{2\pi {\textrm{i}}\eta _{{\mathfrak {j}}_2}}$ and $e^{2\pi {\textrm{i}}\eta _{{\mathfrak {j}}_1}}$ if ${\mathfrak {C}}_{{\mathfrak {j}}_1}$ (resp. ${\mathfrak {C}}_{{\mathfrak {j}}_2}$) is the chamber corresponding to the network on the left (resp. right) in the upper row. The rules describing changes of the network in the other pair of pants are obtained by obvious replacements

### Comparison

In this section we have associated FN-type coordinates to each chamber ${\mathfrak {C}}_{{\mathfrak {i}},{\mathfrak {j}}}$ with ${\mathfrak {i}},{\mathfrak {j}}={{\mathfrak {1}}},{{\mathfrak {2}}},{{\mathfrak {3}}}$. According to the discussion in Sect. 6 each of these coordinates defines a generalised theta series expansion. Let us recall that each chamber ${\mathfrak {C}}_{{\mathfrak {i}},{\mathfrak {j}}}$ there corresponds to a toric diagram from which we can calculate the topological string partitions using the topological vertex. It is now straightforward to check that the results of the topological vertex computations agree precisely with the coefficient functions in the generalised theta series expansions, chamber by chamber.

In the discussions above we had observed some residual freedom in the definition of the FN-type coordinates left by abelianisation. In the case of Molecule II discussed in Section (8.3) this freedom is represented by the factor $\frac{d_2}{d_2'd_3d_3'}$ in (8.6). One should note, however, that we had observed in Sect. 6 that only a rather small subset of the possible choices of coordinates $(\sigma ,\eta )$ can appear in series expansions of tau-functions of generalised theta series type. The discussions in Sect. 6.3 or [CLT, Section 3.3] show that $\frac{d_2}{d_2'd_3d_3'}$ has to be periodic in $\sigma$, severely restricting the left-over freedom. The choice adopted above is distinguished by the property that the functions $N_i$, $i=1,2,3,s$ representing the normalisation of the tau-functions are *real* for real values of the arguments. The freedom which remains after having imposed all these requirements is inessential. This will be further supported by the relation with the exact WKB method discussed in [CLT].

## Summary and Outlook

### The result

To conclude, let us formulate the resulting picture in a way that suggests various generalisations. Our results amount to a reconstruction of the topological string partition functions from the quantum curve for certain degenerating families $C_z$ of base curves parameterised by a complex number *z* which controls the degeneration occurring for $z\rightarrow 0$. This region of the moduli space of the complex structures of $\Sigma _{u,z}$ is related by mirror symmetry to a region in the extended Kähler moduli space which can be represented as the scaling limit of the Kähler moduli space of the toric CY discussed in Sect. 2. It admits a chamber decomposition with chambers separated by walls associated to flop transitions. The sequel [CLT] will propose a natural extension of this picture to the whole moduli space of quantum curves.

The quadratic differential $q_\lambda (x)$ appearing in the equation defining the quantum curve,

9.1 $$\begin{aligned} (\lambda ^2\partial _x^2+q_\lambda (x))\chi _\pm (x)=0, \end{aligned}$$

is defined for given monodromy data $\mu =\mu (\sigma ,\tau )$ through the Riemann–Hilbert correspondence. From the solutions $\chi _\pm$ one may construct the tau-functions ${{\mathcal {T}}}(\sigma ,\tau ;z)$ as the Fredholm determinants of an integral operator canonically associated to $\chi _\pm$. The tau-functions ${{\mathcal {T}}}(\sigma ,\tau ;z)$ admit an expansion in powers of *z* around the degeneration point $z=0$. By multiplying the tau-functions with suitable monodromy-dependent normalisation factors $N(\sigma )$ one may define free fermion partition functions admitting generalised theta series expansions,

9.2 $$\begin{aligned} {{\mathcal {Z}}}(\sigma ,\tau ;z):=N(\sigma ){{\mathcal {T}}}(\sigma ,\tau ;z)=\sum _{n\in {{\mathbb {Z}}}}e^{in\tau }{{\mathcal {G}}}_N(\sigma +n;z). \end{aligned}$$

Abelianisation gives a natural way to fix the normalisation factors $N(\sigma )$ depending on the choice of a chamber in the extended Kähler moduli space of the local CY. The coefficient functions ${{\mathcal {G}}}_N(\sigma ;z)$ appearing in the expansions (9.2) have been found to be equal to the topological string partition functions, chamber by chamber.

It may look surprising that there is an essentially unambiguous way to construct the partition functions from the quantum curve. The key ingredients fixing the ambiguities are (i) integrability controls possible quantum corrections to the quantum curve (see Sect. 3), (ii) abelianisation provides a canonical way to associate FN-type coordinates to the parts of the moduli space of quantum curves characterised by real Kähler moduli, chamber by chamber, and (iii) a one-to-one correspondence between choices of FN-type coordinates and normalisations for the free fermion partition function admitting generalised theta series expansions. In the sequel [CLT] the role of abelianisation will be taken over by the exact WKB expansion for the quantum curve, which is natural in view of the close relation between exact WKB and abelianisation discussed in [HK].

The generalisation to the case of $C=C_{0,n}$ is straightforward. The variables $(\sigma ,\tau )$ get replaced by tuples $({\underline{\sigma }},{\underline{\tau }})$ where ${\underline{\sigma }}=(\sigma _1,\dots ,\sigma _{n-3})$, and ${\underline{\tau }}=(\tau _1,\dots ,\tau _{n-3})$, and *z* gets similarly replaced by ${\textbf{z}}=(z_1,\dots ,z_{n-3})$. Cases like higher genus surfaces $C=C_{g,n}$ or surfaces with irregular singularities^15^ are certainly within reach. The generalisation to covers of higher degree should be very interesting.

### Role of integrable structures

A source of motivation for our proposal has been the relation between the free fermion partition function at $\lambda =0$ (3.1), and the Hitchin integrable system, established by the identification of the variables $({\textbf{a}},{\underline{\vartheta }})$ as action-angle variables of the Hitchin integrable system. It is shown in [CLT] that (3.1) is recovered in the limit $\lambda \rightarrow 0$.

It furthermore seems intriguing to observe that the dependence on both $({\underline{\sigma }},{\underline{\tau }})$ and ${\textbf{z}}$ appears to be controlled by the integrable structures of the problem, as can be expressed by the equations

9.3a $$\begin{aligned}&{\partial _{z_r}{{\mathcal {T}}}_N({\underline{\sigma }},{\underline{\tau }};{\textbf{z}})} =H_r \,{{{\mathcal {T}}}_N({\underline{\sigma }},{\underline{\tau }};{\textbf{z}})}, \end{aligned}$$

9.3b $$\begin{aligned}&{e^{\partial _{\sigma _k}}{{\mathcal {T}}}_N({\underline{\sigma }},{\underline{\tau }};{\textbf{z}})}= e^{-{\textrm{i}}\tau _k}\,{{{\mathcal {T}}}_N({\underline{\sigma }},{\underline{\tau }};{\textbf{z}})}. \end{aligned}$$

The factors $H_r$ appearing on the right hand side of (9.3a) are defined through the Riemann–Hilbert correspondence as functions $H_r=H_r({\underline{\sigma }},{\underline{\tau }};{\textbf{z}})$. The definition of the coordinate $\tau _k$ appearing on the right of (9.3b), on the other hand, is unambiguously fixed by using the solutions $\chi _\pm (x)$ obtained by Borel summation in the definition of coordinates described in Sects. 5.1.

While (9.3a) is the definition of the isomonodromic tau-function through a solution to the Schlesinger equations, the difference equations (9.3b) are associated to the integrable structure of ${{\mathcal {M}}}_{\textrm{flat}}(C_{0,n})$ manifested in the Fenchel-Nielsen type coordinates, allowing one to regard the coordinates ${\underline{\sigma }}$ as action-variables, and ${\underline{\tau }}$ as angle coordinates, together forming a system of Darboux coordintates for the natural symplectic structure on ${{\mathcal {M}}}_{\textrm{flat}}(C_{0,n})$. Equations (9.3b) indicate that the integrable structure of ${{\mathcal {M}}}_{\textrm{flat}}(C_{0,n})$ expressed through the Darboux coordinates $({\underline{\sigma }},{\underline{\tau }})$ can be regarded as a deformation of the integrable structure of the Hitchin system made manifest through the definition of the action-angle coordinates $({\textbf{a}},{\underline{\vartheta }})$.

It is clear that equation (9.3b) severely restricts the dependence of ${{\mathcal {T}}}_N({\underline{\sigma }},{\underline{\tau }};{\textbf{z}})$ on $({\underline{\sigma }},{\underline{\tau }})$, and therefore the choice of the normalisation factors left undetermined by the definition (9.3a) of the isomonodromic tau-function.

### Perspectives

Having given a precise *analytic* characterisation of the topological string partition function may also shed light on what remains to be done to make other approaches fully effective. We will here mention a few possible directions, referring to [CLT] for a discussion of some further directions.

#### Topological recursion

Topological recursion provides a systematic approach to the expansion of the topological string partition functions in powers of $\lambda$, see [Ey] for a review and further references. However, it would be good to know which initial conditions characterise the topological string partition functions for local CY of class $\Sigma$, and to what extend one can reconstruct the non-perturbative answer from the formal series in $\lambda$ defined by the topological recursion.

The topological recursion has recently been used in [IKT] to compute the partition functions associated to three-punctured spheres $C_{0,3}$. The result agrees with the expansions for $\lambda \rightarrow 0$ of the functions $N_{i}(a_3/\lambda ,a_2/\lambda ,a_1/\lambda )$, $i=1,2,3,s$, defined in Sect. 7, as can be checked using the asymptotic expansion of the *G*-function for large arguments. The expansions do not depend on the chamber, whereas the dependence of the exact result on the choice of chamber is exactly the one described in this paper. This is explained in more detail in [CPT19, Section 7.3].

#### Matrix models

Matrix models [DV02, DV09] can potentially give answers for the values of the topological string partition functions which are non-perturbative in $\lambda$ but restricted to a lattice in the set of allowed Kähler parameters defined by the integrality of the numbers of integrations. The precise answer will depend on the choice of integration contours, in general. Interesting questions are (i) which choice of integration contours reproduces the non-perturbative partition functions defined in our paper and (ii) if there is a canonical way to reconstruct the full partition functions from the functions on the lattices in the set of Kähler parameters defined by the matrix models. Partial results concerning the first question (i) have been obtained in [CDV].

#### Topological vertex and beyond

It is not known if the series defined by the topological vertex formalism are convergent, in general, see however [FM] for recent results allowing to prove the convergence in some cases. For the theories of class $\Sigma$ one may deduce analyticity of the topological string partition functions with the help of the Fredholm determinant representations discussed in this paper.

It is worth noting, however, that the class of theories for which the approach taken in this paper suggests an answer includes many cases for which it is not known how to represent the local CY as limits of toric CY. This will be the case for coverings of surfaces *C* of higher genus and the so-called Sicilian quivers. It should be possible to generalise our approach to arrive at precise predictions for this class of local CY for which not much seems to be known at present.

#### ${\mathcal {N}} = 1$ theories

Going beyond the various applications of topological string theory to the study of ${{\mathcal {N}}}=2$ supersymmetric field theories studied in the literature, there should also be interesting applications to field theories having only ${\mathcal {N}} = 1$ supersymmetry in four dimensions. Intriligator and Seiberg have made a first step in this direction by generalizing the Seiberg-Witten theory [IS]. Using their work we can characterize the low-energy physics of field theories with an abelian Coulomb branch by spectral curves in a way which is somewhat analogous to the cases with ${{\mathcal {N}}}=2$ supersymmetry. It would be very interesting if the technology developed in this paper could be generalized to predict partition functions for ${\mathcal {N}} = 1$ theories for which only very few tools exist, see [CPTY, MP, BP] for some previous work in this direction.

## Grassmannians and Sato–Segal–Wilson Tau-Function

The construction of free fermion partition function proposed in [DHS] was based on the theory of infinite Grassmannians pioneered in [Sa, SW]. We will here compare our formulation to the one used in [DHS].

### Grassmannians and tau-functions

#### Infinite Grassmannians

Let ${{\mathcal {H}}}=L^2(S^1,{\mathbb {C}}^N)$, where $S^1$ will often be identified with the equator of ${\mathbb {P}}^1$. Elements of ${{\mathcal {H}}}$ will be represented as vectors having functions on $S^1$ in each of their *N* components. We have ${{\mathcal {H}}}={{\mathcal {H}}}_+\oplus {{\mathcal {H}}}_-$, where the functions in ${{\mathcal {H}}}_+$ (${{\mathcal {H}}}_-$) can be continued analytically inside $S^1$ (outside of $S^1$ and vanish at infinity). The Segal-Wilson Grassmannian ${\textrm{Gr}}({{\mathcal {H}}})$ is the set of all closed subspaces *W* of ${{\mathcal {H}}}$ which are the images of embeddings ${{\textsf{w}}}_+\oplus {{\textsf{w}}}_-:{{\mathcal {H}}}_+\rightarrow {{\mathcal {H}}}_+\oplus {{\mathcal {H}}}_-$ such that ${{\textsf{w}}}_+$ is a Fredholm operator, and ${{\textsf{w}}}_-$ is a compact operator. A subspace *W* in ${\textrm{Gr}}({{\mathcal {H}}})$ is spanned by the columns of the rectangular matrix $${{\textsf{w}}}=({\begin{matrix}{{\textsf{w}}}_+\\ {{\textsf{w}}}_-\end{matrix}})$$ called a frame of *W*. Frames related by right multiplication with elements of the group ${{\mathcal {C}}}$ of invertible operators ${{\textsf{h}}}:{{\mathcal {H}}}_+\rightarrow {{\mathcal {H}}}_+$ differing from the identity ${\textbf{1}}$ by a trace class operator describe the same points in ${\textrm{Gr}}({{\mathcal {H}}})$.

A frame is called admissible if ${{\textsf{w}}}_+-{\textbf{1}}$ is a trace-class operator on ${{\mathcal {H}}}_+$. We restrict attention to subspaces *W* having admissible frames. A frame for a space *W* can be transformed into an admissible frame if ${{\textsf{w}}}_+$ is invertible,

A.1 $$\begin{aligned} \bigg (\begin{matrix} {{\textsf{w}}}_+ \\ {{\textsf{w}}}_- \end{matrix}\bigg )= \bigg (\begin{matrix} {\textbf{1}} \\ {{\textsf{w}}}_-^{}{{\textsf{w}}}_+^{-1} \end{matrix}\bigg )\cdot {{\textsf{w}}}_+ =: \bigg (\begin{matrix} {\textbf{1}} \\ {\textsf{A}} \end{matrix}\bigg )\cdot {{\textsf{w}}}_+. \end{aligned}$$

This means that the space *W* can be described as the graph of the operator ${\textsf{A}}:{{\mathcal {H}}}_+\rightarrow {{\mathcal {H}}}_-$, ${\textsf{A}}:={{\textsf{w}}}_-^{}{{\textsf{w}}}_+^{-1}$. Such a space *W* is called transverse to ${{\mathcal {H}}}_-$.

A natural line bundle on the Segal-Wilson Grassmannian ${\textrm{Gr}}({{\mathcal {H}}})$ is the dual of the determinant bundle ${\textrm{Det}}^*$, which can be represented by pairs $({{\textsf{w}}},\lambda )$ with $({{\textsf{w}}},\lambda )$ and $({{\textsf{w}}}',\lambda ')$ considered to be equivalent iff ${{\textsf{w}}}'={{\textsf{w}}}{{\textsf{t}}}$ and $\lambda '=\lambda {\textrm{det}}({{\textsf{t}}})$ for ${{\textsf{t}}}\in {{\mathcal {C}}}$. ${\textrm{Det}}^*$ has a canonical section $\sigma$ represented by the pairs $({{\textsf{w}}},{\textrm{det}}({{\textsf{w}}}_+))$.

#### Definition of Sato–Segal–Wilson tau-functions

Let $\Gamma$ be the group of continuous maps $S^1\rightarrow {\textrm{GL}}(N)$, regarded as multiplication operators on ${{\mathcal {H}}}$. The subgroups $\Gamma _+$ and $\Gamma _-$ are represented by real analytic functions *f* which extend holomorphically inside the unit circle satisfying $f(0)=1$ and outside of the unit circle with $f(\infty )=1$, respectively. Noting that the multiplication by $g^{-1}\in \Gamma _+$ is represented on ${{\mathcal {H}}}={{\mathcal {H}}}_+\oplus {{\mathcal {H}}}_-$ by a matrix of the form $$ \left( {\begin{matrix} {\textsf{a}} &{} {\textsf{b}}\\ 0&{}{\textsf{d}} \end{matrix}}\right) $$ one may define the tau-function $\tau _W^{}(g)$ for *W* transverse to ${{\mathcal {H}}}_-$ as a function on $\Gamma _+$ by setting

A.2 $$\begin{aligned} \tau _W^{}(g)=\frac{\sigma (g^{-1}W)}{g^{-1}\sigma (W)}, \end{aligned}$$

where $g^{-1}\sigma$ is the natural action of $g^{-1}$ on sections of ${\textrm{Det}}^*$, see [SW] for details. It is not hard to see that the function $\tau _W$ can be represented as the Fredholm determinant

A.3 $$\begin{aligned} \tau _W^{}(g)={\textrm{det}}_{{{\mathcal {H}}}_+}^{}({\textsf{1}}+{\textsf{B}}_g{\textsf{A}}),\qquad {\textsf{B}}_g={\textsf{a}}^{-1}{\textsf{b}}. \end{aligned}$$

This construction can be applied in particular to the case $W=\Psi ^{-1}\cdot {{\mathcal {H}}}_+$, with $\Psi$ being the fundamental solution matrix of holonomic ${{\mathcal {D}}}$-modules which can be analytically continued outside of $S^1$ and is twice differentiable on $S^1$. In this way we get a natural way to associate points in ${\textrm{Gr}}({{\mathcal {H}}})$ to ${{\mathcal {D}}}$-modules.

### Free fermion states associated to points in the infinite Grassmannian

We are now going to show that the tau-function defined in (A.3) can be represented as a matrix element in the fermionic Fock space. This connection was part of the motivation for the proposal made in [DHS] that the free fermion partition functions relevant for topological string theory can be defined using the above-mentioned connection between ${{\mathcal {D}}}$-modules and points in ${\textrm{Gr}}({{\mathcal {H}}})$. The approach described in the main text realises these ideas for the case of our interest.

The spaces $W\in {\textrm{Gr}}({{\mathcal {H}}})$ we are interested in are spanned by the columns of $${{\textsf{w}}}=({\begin{matrix}{{\textsf{w}}}_+\\ {{\textsf{w}}}_-\end{matrix}})$$ with ${{\textsf{w}}}_+$ invertible. Such spaces are specified by the graphs of the operators ${{\textsf{A}}}={{\textsf{w}}}_-^{}{{\textsf{w}}}_+^{-1}$. Let the operator ${{\textsf{A}}}:{{\mathcal {H}}}_+\rightarrow {{\mathcal {H}}}_-$ be represented by matrices $A_{kl}$ with respect to the bases ${{\mathcal {B}}}_+$ and ${{\mathcal {B}}}_-$ for the Hilbert spaces ${{\mathcal {H}}}_+$ and ${{\mathcal {H}}}_-$, respectively, where ${{\mathcal {B}}}_+=\{{e}_sz^{l},s=1,\dots ,N;l=0,1,\dots \}$, and ${{\mathcal {B}}}_-=\{{e}_sz^{-k},s=1,\dots ,N;k=1,2\dots \}$, with $\{e_s,s=1,\dots ,N\}$ being the canonical basis of ${{\mathbb {C}}}^N$. We may then define a vector ${\mathfrak {f}}_A^{}\in {{\mathcal {F}}}$ as

A.4 $$\begin{aligned}&{\mathfrak {f}}_A^{}:={\textsf{U}}_A^{}\cdot {\mathfrak {f}}_{0}^{},\qquad {\textsf{U}}_A= \exp \bigg (-\sum _{k>0}\sum _{l\ge 0} \psi _{-k}\cdot A_{kl}\cdot {\bar{\psi }}_{-l}\bigg ) . \end{aligned}$$

A function $\Psi (y):S^1\rightarrow {\textrm{GL}}(N,{{\mathbb {C}}})$ analytic outside of $S^1$ and twice-differentiable on $S^1$ defines a multiplication operator on ${{\mathcal {H}}}$ allowing us to define the operator

A.5 $$\begin{aligned} {\textsf{A}}_\Psi =\Pi _-\,\Psi ^{-1}\,\Pi _+\,\Psi \,\Pi _+, \end{aligned}$$

where $\Pi _\pm :{{\mathcal {H}}}\rightarrow {{\mathcal {H}}}_\pm$ are the canonical projections. The operator $\Pi _-\,\Psi ^{-1}\,\Pi _+$ is trace-class [SW, Propositon 2.3], from which it follows that ${\textsf{A}}_\Psi$ is trace-class as well. One may represent ${{\textsf{A}}}_\Psi$ as an integral operator

A.6 $$\begin{aligned} ({\textsf{A}}_\Psi f)(x)=\frac{{\textsf{i}}}{2\pi } \int _{{{\mathcal {C}}}}{dy}\,\frac{(\Psi (x))^{-1}\Psi (y)}{x-y}f(y). \end{aligned}$$

From (A.6) it is clear that the matrices representing ${\textsf{A}}_\Psi$ with respect to this basis are defined using (4.11) and (4.8). We recover the construction used in Sect. 4.1 to define free fermion states ${\mathfrak {f}}_\Psi \in {{\mathcal {F}}}$ from solutions to the Riemann–Hilbert problem.

An operator ${{\textsf{B}}}:{{\mathcal {H}}}_-\rightarrow {{\mathcal {H}}}_+$ represented by matrices $B_{lk}$ can in a similar way be used to define

A.7 $$\begin{aligned}&{\mathfrak {f}}_B^{*}:= {\mathfrak {f}}_{0}^{*}\cdot {\textsf{V}}_B^{},\qquad {\textsf{V}}_B^{}=\exp \bigg (-\sum _{l\ge 0}\sum _{k>0} {\bar{\psi }}_{l}\cdot B_{lk}\cdot {\psi }_{k}\bigg ). \end{aligned}$$

One may again associate such operators in particular to functions $\Psi (y)$ analytic inside of $S^1$ and twice differentiable on $S^1$. Representing the multiplication operator $(\Psi ^{\textrm{out}})^{-1}$ with respect to ${{\mathcal {H}}}\simeq {{\mathcal {H}}}_+\oplus {{\mathcal {H}}}_-$ in the form $$\left( {\begin{matrix} {\textsf{a}} &{} {\textsf{b}}\\ 0&{}{\textsf{d}}\end{matrix}}\right) $$, allows us to define a trace-class operator ${{\textsf{B}}}_{\Psi }:{{\mathcal {H}}}_-\rightarrow {{\mathcal {H}}}_+$, ${{\textsf{B}}}_{\Psi }={\textsf{a}}^{-1}{\textsf{b}}$ and a state ${\mathfrak {f}}_\Psi ^*\in {{\mathcal {F}}}^*$ in a way which is analogous to the definition of ${{\textsf{A}}}_{\Psi }$ and ${\mathfrak {f}}_\Psi$ given above.

The Fredholm determinants representing the Sato–Segal–Wilson tau-functions $\tau _W^{}(g)$ via (A.3) can now be represented as matrix elements in the free fermion Fock-space,

A.8 $$\begin{aligned} {\textrm{det}}_{{{\mathcal {H}}}_+}^{}({\textsf{1}}+{\textsf{B}}_g{\textsf{A}})=\langle \,{\mathfrak {f}}_{B_g}^*,\,{\mathfrak {f}}_A^{}\,\rangle , \end{aligned}$$

the next subsection contains a self-contained proof of the identity (A.8).

### Determinant representation of fermionic matrix elements

Our goal in this subsection is to prove the identity

A.9 $$\begin{aligned} {\textrm{det}}_{{{\mathcal {H}}}_+}^{}({\textsf{1}}+{\textsf{B}}{\textsf{A}})=\langle \,{\mathfrak {f}}_B^*,\,{\mathfrak {f}}_A^{}\,\rangle , \end{aligned}$$

where ${\textsf{A}}:{{\mathcal {H}}}_+\rightarrow {{\mathcal {H}}}_-$ and ${\textsf{B}}:{{\mathcal {H}}}_-\rightarrow {{\mathcal {H}}}_+$ are trace-class operators, and ${\mathfrak {f}}_A^{}\in {{\mathcal {F}}}$ and ${\mathfrak {f}}_B^{*}\in {{\mathcal {F}}}^*$ are the states defined from ${{\textsf{A}}}$ and ${{\textsf{B}}}$ in (A.4) and (A.7), respectively. Identities like (A.9) are probably known, but we did not find a convenient reference for the proof.

It will be useful to represent the elements of ${{\mathcal {H}}}=L^2(S^1,{\mathbb {C}}^N)$ using an isomorphism ${{\mathcal {H}}}\simeq L^2(S^1,{\mathbb {C}})$ called blending. Introducing the canonical basis $(e_1,\dots ,e_N)$ of ${{\mathbb {C}}}^N$, one may map

A.10 $$\begin{aligned}&L^2(S^1,{\mathbb {C}}^N)\ni f(x)=\sum _{n\in {{\mathbb {Z}}}}\sum _{k=1}^N f_{n,k}x^{-n}e_k, \quad \mapsto \quad g(x)=\sum _{m\in {{\mathbb {Z}}}}g_m x^{-m}\in L^2(S^1,{\mathbb {C}}),\nonumber \\&\text {where}\quad g_{nN+k}:=f_{n,k}, \quad k=1,\dots ,N,\quad n\in {{\mathbb {Z}}}. \end{aligned}$$

Let us next notice that

A.11 $$\begin{aligned} {\textrm{det}}_{{{\mathcal {H}}}_+}^{}({\textsf{1}}+{\textsf{B}}{\textsf{A}})= {\textrm{det}}_{{{\mathcal {H}}}}^{}(1+{\textsf{D}}), \qquad {\textsf{D}}= \bigg (\begin{matrix} 0 &{} -{\textsf{B}} \\ {\textsf{A}} &{} \;\,0\end{matrix} \bigg ). \end{aligned}$$

Using the blending isomorphism we may represent ${\textsf{D}}$ as a ${{\mathbb {Z}}}\times {{\mathbb {Z}}}$-matrix *D*. The determinant ${\textrm{det}}_{{{\mathcal {H}}}}^{}(1+{\textsf{D}})$ can then be expanded as

A.12 $$\begin{aligned} {\textrm{det}}_{{{\mathcal {H}}}}^{}(1+{\textsf{D}})=\sum _{{{\mathbb {S}}\subset {{\mathbb {Z}}},\, |{\mathbb {S}}|<\infty }} {\textrm{det}}({D}_{\mathbb {S}}), \end{aligned}$$

where ${D}_{\mathbb {S}}$ is the matrix obtained from *D* by deleting all rows and columns in ${\mathbb {Z}}{{\setminus }} {\mathbb {S}}$.

We may further decompose ${\mathbb {S}}\subset {\mathbb {Z}}$ into two sets ${\mathbb {P}}={\mathbb {S}}\cap {\mathbb {Z}}^{\ge 0}$ and ${\mathbb {H}}={\mathbb {S}}\cap {\mathbb {Z}}^{< 0}$. The block structure of ${\textsf{D}}$ implies that ${\mathbb {P}}$ and ${\mathbb {H}}$ have the same cardinality. Using the blending isomorphism we may represent ${\textsf{A}}$ as a ${{\mathbb {Z}}}^{\ge 0}\times {{\mathbb {Z}}}^{<0}$-matrix $A^b$, and ${\textsf{B}}$ as a ${{\mathbb {Z}}}^{< 0}\times {{\mathbb {Z}}}^{\ge 0}$-matrix $B^b$. Due to the block structure of ${\textsf{D}}$ one may factorise ${\textrm{det}}({D}_{\mathbb {S}})$ as

A.13 $$\begin{aligned} {\textrm{det}}({D}_{\mathbb {S}})= {\textrm{det}}({B}_{{\mathbb {H}}{\mathbb {P}}}) {\textrm{det}}({A}_{{\mathbb {P}}{\mathbb {H}}}), \end{aligned}$$

where ${A}_{{\mathbb {P}}{\mathbb {H}}}$ is obtained from $A^b$ by deleting all rows with indices not contained in ${\mathbb {P}}$ and all columns having indices not in ${\mathbb {H}}$, with ${B}_{{\mathbb {H}}{\mathbb {P}}}$ defined in an analogous way.

The formula following by inserting (A.13) into (A.12) can be directly compared to the representation of $\langle \,{\mathfrak {f}}_B^{*},\,{\mathfrak {f}}_A^{}\,\rangle$ in terms of the expansion

A.14 $$\begin{aligned} \langle \,{\mathfrak {f}}_B^{*},\,{\mathfrak {f}}_A^{}\,\rangle = \sum _{\imath \in {{\mathcal {I}}}}\langle \,{\mathfrak {f}}_B^{*},\,{\mathfrak {f}}_{\imath }^{}\,\rangle \langle \,{\mathfrak {f}}_{\imath }^*,\,{\mathfrak {f}}_A^{}\,\rangle , \end{aligned}$$

with $\{{\mathfrak {f}}_{\imath }^{};\imath \in {{\mathcal {I}}}\}$ and $\{{\mathfrak {f}}_{\imath }^{*};\imath \in {{\mathcal {I}}}\}$ being bases for ${{\mathcal {F}}}$ and ${{\mathcal {F}}}^*$, respectively, such that $\langle \,{\mathfrak {f}}_\jmath ^{*},\,{\mathfrak {f}}_{\imath }^{}\,\rangle =\delta _{\imath ,\jmath }$.

The blending isomorphism relates N-component vectors $\psi (z)$, ${\bar{\psi }}(z)$ on a punctured disc to fermions $\phi (z)$, ${\bar{\phi }}(z)$ on an N-fold cover of the punctured disc with modes being related as $\phi _{nN+s-1}=\psi _{s,n}$, ${{\bar{\phi }}}_{nN+s}={\bar{\psi }}_{s,n}$. The Fock-space ${{\mathcal {F}}}$ thereby gets an alternative representation as Fock space of a single species of free fermions. A useful pair of dual bases for ${{\mathcal {F}}}$ and ${{\mathcal {F}}}^*$ can be generated from the vectors

A.15 $$\begin{aligned} {\mathfrak {f}}_{{\mathbb {P}}{\mathbb {H}}}^*= {\mathfrak {f}}_0^*\;\phi _{n_p}\dots \phi _{n_1}{\bar{\phi }}_{m_1}\dots {\bar{\phi }}_{m_h}, \qquad {\mathfrak {f}}_{{\mathbb {H}}{\mathbb {P}}}^{}= \phi _{-m_h}\dots \phi _{-m_1}{\bar{\phi }}_{-n_1}\dots {\bar{\phi }}_{-n_p}\,{\mathfrak {f}}_0^{},\qquad \end{aligned}$$

associated to the finite sets ${\mathbb {P}}=\{n_1,\dots ,n_p\}\subset {{\mathbb {Z}}}^{\ge 0}$ and ${\mathbb {H}}=\{-m_1,\dots ,-m_q\}\subset {{\mathbb {Z}}}^{<0}$.

It remains to prove the identities

A.16a $$\begin{aligned}&\langle \,{\mathfrak {f}}_{{\mathbb {P}}{\mathbb {H}}}^*\,,\,{\mathfrak {f}}_A^{}\,\rangle =(-)^{p}\,{\textrm{det}}({A}_{{\mathbb {P}}{\mathbb {H}}}), \end{aligned}$$

A.16b $$\begin{aligned}&\langle \,{\mathfrak {f}}_B^{*}\,,\,{\mathfrak {f}}_{{\mathbb {H}}{\mathbb {P}}}^{}\,\rangle =(-)^{p}\,{\textrm{det}}({B}_{{\mathbb {H}}{\mathbb {P}}}). \end{aligned}$$

To prove (A.16a) one may use the identities

A.17 $$\begin{aligned} {{\bar{\phi }}}_{k}\,{\mathfrak {f}}_A^{}=-\sum _{l\ge 0}A_{kl}^b{\bar{\phi }}_{-l}\,{\mathfrak {f}}_A^{}, \end{aligned}$$

following directly from the definition of ${\mathfrak {f}}_A^{}$, allowing us to calculate

$$\begin{aligned} \langle \,{\mathfrak {f}}_{{\mathbb {P}}{\mathbb {H}}}^*\,,\,{\mathfrak {f}}_A^{}\,\rangle&= \langle \, {\mathfrak {f}}_0^*\,,\,\phi _{n_p}\dots \phi _{n_1}{\bar{\phi }}_{m_1}\dots {\bar{\phi }}_{m_p}\, {\mathfrak {f}}_A\rangle \\&\!\!\!\overset{(A.17)}{=}-(-)^{p-1}\sum _{m} A_{m_1m}^b \langle \,{\mathfrak {f}}_0\,,\,\phi _{n_p}\dots \phi _{n_1}{\bar{\phi }}_{m_2}\dots {\bar{\phi }}_{m_p} {\bar{\phi }}_{-m}\, {\mathfrak {f}}_A^{}\rangle \\&=-\sum _{l=1}^p A_{m_1n_l}^b(-)^{l-1}\langle \,{\mathfrak {f}}_0^*\,,\,\phi _{n_p}\dots \phi _{n_{l+1}}\phi _{n_{l-1}}\dots \phi _{n_1}{\bar{\phi }}_{m_2}\dots {\bar{\phi }}_{m_p} \,{\mathfrak {f}}_A^{}\rangle \end{aligned}$$

Using this identity recursively, and comparing the result with Laplace’s formula for ${\textrm{det}}({A}_{{\mathbb {P}}{\mathbb {H}}})$ one gets the identity (A.16a). The proof of (A.16b) is completely analogous.

## Footnotes

1 This terminology can be motivated in two ways. Weighted sums over functions with arguments shifted by lattice translations are sometimes called theta series in the mathematical literature. In [CLT] it is shown, on the other hand, that ordinary theta functions can be recovered from $Z_{\textrm{ff}}(\xi ,t;\lambda )$ in the limit $\lambda \rightarrow 0$. We may therefore regard the partition functions $Z_{\textrm{ff}}(\xi ,t;\lambda )$ as deformations of ordinary theta functions.

2 The approach of [GHM] considers Fredholm determinants constructed from the quantum curves of toric CY. However, the relation to the Fredholm determinants appearing in our paper is not clear to us.

3 Section 2 in [AKMV] summarises the relevant background on toric geometry in a well-suited form.

4 It is possible that the following results have been derived more directly in the mathematical literature on mirror symmetry, but we did not find a reference where this has been worked out explicitly for the case of our interest.

5 A formal linear combination of oriented paths, not necessarily closed, with integral coefficients.

6 Going back to [Sk], applied to Hitchin systems in [Hu, GNR, Kr02], and reviewed in [T17b, Section 2].

7 A $\lambda$-connection $\nabla _\lambda$ satisfies $\nabla _\lambda f s=\lambda (\partial f)s+f \nabla _\lambda s$ for smooth functions *f* and sections *s* of ${{\mathcal {E}}}$.

8 A review of CFT with a very similar perspective can be found in [T17a].

9 This is reviewed in [T17a] using a very similar formalism as used in our paper.

10 The main idea is simple [Mo]: Consider the expansion of the fermion two point function $G_{\Psi }(x,y)$ around $x=y$. Using (4.15) and $\partial _x\Psi =A\Psi$ one may observe that the trace part contains ${{\textrm{tr}}} A^2(x)$ at order ${{\mathcal {O}}}(x-y)$. The expansion may also be calculated using the OPE of the fermionic fields where *T*(*x*) appears at the same order. Comparing the resulting expressions yields (4.19). A proof within the formalism used here was outlined in [T17a].

11 The dependence on ${\underline{\theta }}=(\theta _1,\dots ,\theta _4)$ is temporarily suppressed in the notations.

12 Comparing (6.2) with the results of [GIL, ILT, GL16] it may be helpful to take the discussion in Sects. 6.2 and 6.3 into account. Appendix B in [CLT] explains in detail how to derive (6.10) from the main result of [ILT]. Formula (6.2) is equivalent, and more directly related to the discussion in Sect. 5.

13 Some of the older references had incorrectly claimed that the topological string partition functions are invariant under flop transitions.

14 These figures have been plotted using the Mathematica package [Npl].

15 See [BGT16, BLMST, BGT17] for similar results obtained by different approaches in some cases with irregular singularities.
